# Research Progress of Insulin-like Growth Factor-1 in Major Neurodegenerative Diseases

**DOI:** 10.3390/ijms27219696

**Published:** 2026-10-29

**Authors:** Yueyan Sun, Jiaqi Xia

**Affiliations:** 1 School of Clinical Medical Sciences, Jiamusi University, Jiamusi 154007, China; 23190150123@stu.jmsu.edu.cn; 2 School of Basic Medical Sciences, Jiamusi University, Jiamusi 154007, China

**Keywords:** neurodegenerative diseases, IGF-1, Alzheimer’s disease, Parkinson’s disease

## Abstract

Major neurodegenerative diseases (NDDs) represent a large category of disorders characterized by the progressive loss of structure, function, or eventual death of neuronal cells in the brain and spinal cord. Current conventional therapeutic strategies for NDDs primarily focus on symptomatic relief. In contrast, IGF-1 is a pleiotropic protein with neurotrophic, synaptogenic, and anti-apoptotic properties, and its neuroprotective potential has been demonstrated in various preclinical models of Alzheimer’s disease, Parkinson’s disease, and other NDDs. However, the clinical efficacy of IGF-1-based interventions—whether for symptomatic relief or disease modification—has not been established in humans. Research on IGF-1 as a therapeutic target for NDDs remains predominantly preclinical, and its translation into effective clinical treatments requires further validation. This narrative review summarizes the mechanisms of action and therapeutic advances of IGF-1 in the progression of NDDs, encompassing the following four aspects: (1) Strategies based on signaling pathway regulation that target key pathways such as IGF-1/PI3K/Akt to reduce neuronal loss, promote synaptogenesis and structural remodeling, correct anomalous tau hyperphosphorylation, and improve amyloid-β (Aβ) clearance impairment. (2) Strategies based on restoring central sensitivity that focus on ameliorating cerebral insulin/IGF-1 resistance and utilizing agents to indirectly restore the neuroprotective function of IGF-1, thereby delaying cognitive decline. (3) Strategies based on delivery systems: in animal models, these employ viral vectors or nanomaterials to achieve targeted brain delivery of IGF-1, effectively promoting the survival of dopaminergic and motor neurons. (4) Future research directions that further elucidate the dynamic evolution of IGF-1 signaling across different disease stages and the development of stage-specific regulatory strategies. This review discusses approaches to restoring central IGF-1 signaling, ameliorating cerebral insulin resistance, and improving brain-targeted delivery, while emphasizing that their therapeutic relevance remains predominantly preclinical and requires validation in human studies.

## 1. Introduction

NDDs are a large class of central nervous system disorders characterized by progressive neuronal loss in specific brain regions and abnormal protein aggregation, mainly including Alzheimer’s disease (AD), Parkinson’s disease (PD), motor neuron disease (MND), and Huntington’s disease (HD), among others. Although distinct pathological phenotypes are observed across different NDDs, they share several core pathogenic mechanisms: disruption of protein homeostasis, including impaired ubiquitin–proteasome and autophagy-lysosome systems, leading to toxic accumulation of misfolded proteins, sustained activation of microglia and astrocytes driving neuroinflammation [1], oxidative stress and mitochondrial dysfunction inducing energy metabolism impairment, and damaged integrity of the blood–brain barrier exacerbating central nervous system microenvironment disturbance. At present, available therapies for NDDs generally face three major problems: inability to effectively block disease progression, inability to halt or slow the ongoing neuronal loss, and most drugs find it difficult to penetrate the blood–brain barrier to reach the target site [2]. Therefore, disease-modifying therapies are urgently needed for NDDs such as AD and many other disorders characterized by protein aggregation [3].

**Figure 1:**
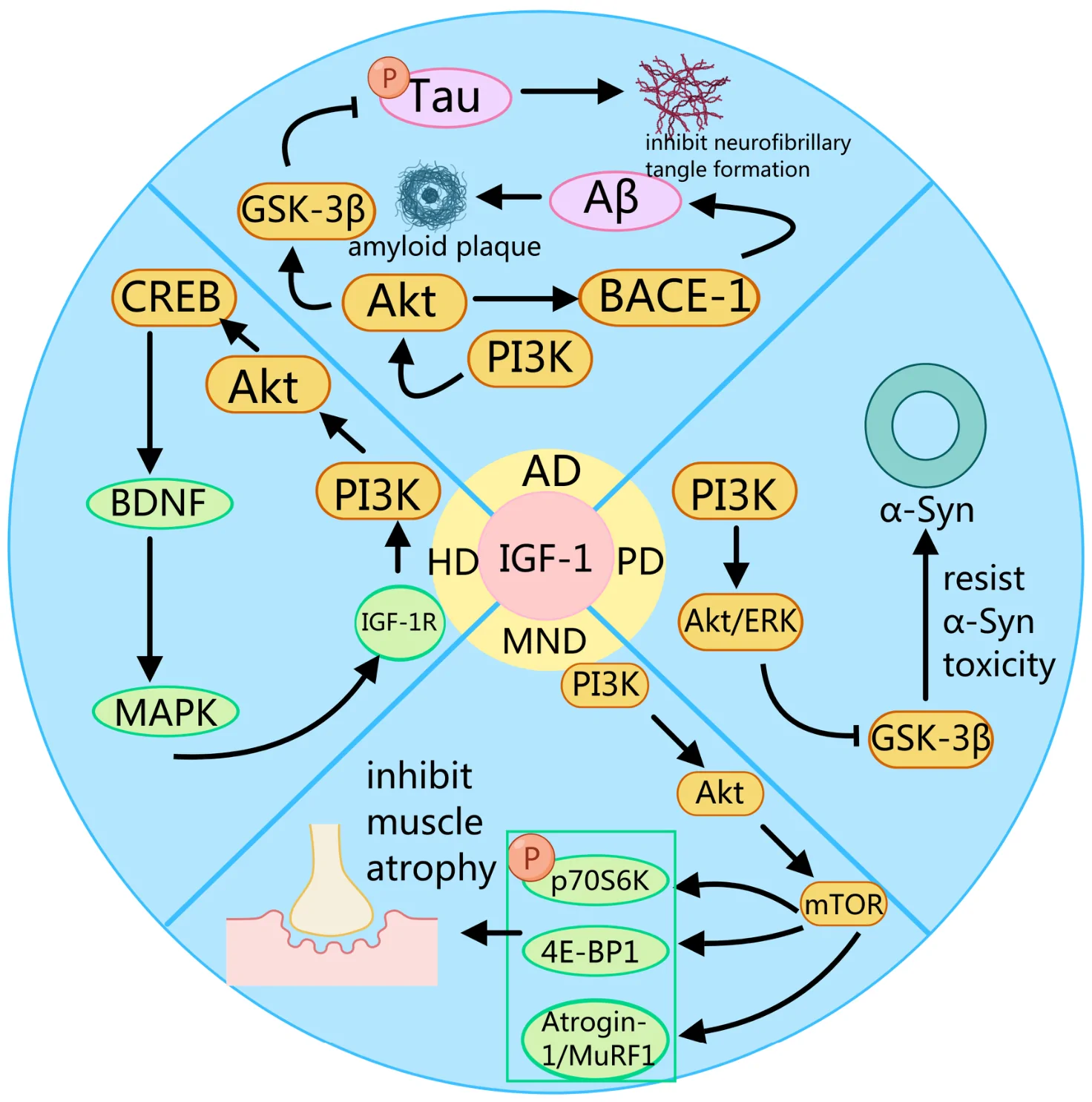
The action mechanisms of IGF-1 in common NDDs. Created with MedPeer (https://www.medpeer.cn).

IGF-1 is a polypeptide hormone composed of 70 amino acid molecules, with a molecular weight of approximately 7649 daltons. It is mainly synthesized by the liver in response to growth hormone (GH) stimulation and is a key downstream factor mediating the stimulatory effects of GH. In addition to endocrine IGF-1 derived from the liver, IGF-1 and its receptors are also locally expressed in multiple regions of the brain, including the hippocampus, cerebral cortex and cerebellum, exerting paracrine and autocrine functions [4,5]. IGF-1 is indispensable in the growth and development of children, the maintenance of adult tissues, and the regulation of metabolic homeostasis, and has a wide range of physiological functions, such as promoting cell proliferation and differentiation [6], regulating metabolic balance [7], and modulating immune responses [8]. The half-life of IGF-1 is closely related to its binding state with binding proteins: free IGF-1 has a half-life of merely 10–12 min, whereas the ternary complex composed of IGF-1, insulin-like growth factor binding proteins 3 (IGFBP-3) and ALS boasts a half-life of 12 to 15 h. Approximately 98% of IGF-1 in the bloodstream binds to IGFBPs, so this hormone requires constant synthesis and release to meet physiological demands [9,10]. IGF-1 signaling pathway, crucial for central nervous system (CNS) development, metabolism, repair, cognition, and emotion regulation, includes IGF-1, IGF-2, IGF-1R, IGF-2R, insulin receptor (IR), and six IGFBPs [11]. In recent years, the role of IGF-1 in the central nervous system has received increasing attention. IGF-1 antagonizes protein toxicity, inhibits neuroinflammation, and improves energy metabolism, playing a key role in protecting synaptic integrity and preventing the progressive loss of neurons. As a potential therapeutic target in this field, IGF-1 holds promise for breaking through the limitations of existing treatments that merely alleviate symptoms. Nevertheless, while preclinical studies have generated abundant supportive evidence, significant obstacles remain in translating these findings into effective clinical treatments. As summarized in Figure 1, preclinical studies indicate that IGF-1 can engage signaling pathways such as PI3K/Akt that are associated with neuronal survival, proteostasis, and synaptic plasticity. Notably, their downregulated expression or impaired signal transduction has emerged as a shared pathological hallmark across multiple NDDs. Kaur and Aran research has confirmed that IGF-1 can reduce Aβ deposition and tau protein over phosphorylation in AD [12], and suggests that abnormal IGF-1 signaling is closely related to the occurrence and development of PD [13]. However, the relationship between IGF-1 and NDDs is far more complex than initially thought. Epidemiological and clinical studies suggest heterogeneous associations between IGF-1/insulin signaling and dementia risk, with adverse outcomes reported at both lower and higher signaling states in different contexts [14]. These findings are compatible with, but do not establish, a unified quantitative U-shaped dose–response relationship. These findings highlight the need for further investigation of IGF-1 signaling across different experimental and disease contexts before specific therapeutic strategies can be established. Additionally, the clinical translation of IGF-1 faces two obstacles: first, although IGF-1 can cross the blood–brain barrier mediated by LRP1/LRP2 receptors, its bioavailability in the brain is extremely low after systemic administration, making it difficult to reach the concentration required for treatment [15]. Second, long-term systemic administration may cause hypoglycemia, edema, and potential tumor-promoting risks [16]. Although several reviews have examined the role of IGF-1 in AD and PD, these accounts remain largely disease-specific; a side-by-side comparison of IGF-1 signaling, its pathological context, and therapeutic strategies across AD, PD, MND and HD is still lacking. More importantly, although IGF-1 has attracted considerable interest as a neurotrophic factor in NDD research, its functions exhibit prominent pathological context dependence, and this contradictory phenomenon requires systematic elucidation. Specifically, IGF-1 can effectively counteract the toxicity of amyloid proteins and α-synuclein by activating the IGF-1R/PI3K/Akt signaling pathway in AD and PD models [17]. However, in HD models with mitochondrial damage, it may lose protective effects or even exacerbate neuronal death due to disturbed energy metabolism. Additionally, in-depth discussions on the dynamic changes of IGF-1 signaling at different disease stages of NDDs are insufficient. Crucially, there is a paucity of discussion regarding the translational potential and clinical therapeutic strategies of IGF-1. Recent research highlights the importance of understanding these signaling networks, as impaired IGF-1 signaling is associated with neurodegeneration [18]. This article aims to systematically review the key mechanisms of IGF-1 in NDDs and explore multiple strategies to break through its transformation bottleneck, with the aim of providing a framework for future mechanistic and translational investigation of IGF-1 in NDDs.

## 2. IGF-1 and NDDs

NDDs are characterized by the progressive dysfunction and loss of specific neuronal populations. Clinically and pathologically, they are commonly grouped by predominant neuronal involvement and core manifestations: ① dementias, exemplified by AD; ② movement disorders, such as PD; ③ MND, including ALS; and ④ other disorders, such as HD. AD is pathologically characterized by extracellular deposition of Aβ forming amyloid plaques, and neurofibrillary tangles induced by hyperphosphorylation of tau protein [19]. PD is defined by the loss of dopaminergic neurons in the substantia nigra, accompanied by aggregation of α-synuclein to form Lewy bodies as its core feature [20,21]. The majority of sporadic amyotrophic lateral sclerosis (ALS) patients (up to 97%) also contain the TDP-43 protein deposited in the neuronal inclusions, which suggests its pivotal role in the ALS pathology [22]. HD features intracellular abnormal aggregation of mutant huntingtin (mHTT) and selective loss of striatal projection neurons and interneurons in the striatum [23].

Despite diverse phenotypes, these conditions share convergent mechanisms, notably impaired protein homeostasis (proteostasis) and progressive neuronal loss, which require deeper mechanistic dissection and therapeutic innovation [24]. As summarized in Table 1, a diverse array of growth factors, including IGF-1, IGF-2, and so on, exert pivotal regulatory effects across NDDs. As a therapeutic target across NDDs, insulin-like IGF-1 exhibits pleiotropic neurotrophic and neuroprotective actions with broad relevance to key pathological processes in AD, PD, MND/ALS, and HD [25]. IGF-1 signaling engages the PI3K/Akt axis in AD, PD, MND, and HD. In AD, disrupted IGF-1 signaling is closely linked to defective Aβ clearance and tau pathology [26]. In PD, IGF--1 supports dopaminergic neuron survival and modulates α-syn toxicity [27]. In MND/ALS, IGF-1 can promote motor neuron viability and may attenuate disease progression [28]. In HD, IGF-1 signaling is implicated in mitigating mutant huntingtin-induced toxicity and preserving striatal neuron integrity [29]. This review synthesizes recent advances, mechanistic insights, and clinical research prospects for targeting IGF-1 in these major neurodegenerative disorders.

**Table 1:** Regulation of NDDs by growth factors via related receptors.

Growth Factors	Main Receptor	Disease	References
NGF	TrkA/p75NTR	AD	[30]
BDNF	TrkB/p75NTR	AD; PD; HD	[31,32,33,34]
GDNF	GFRα1-RET complex	PD; ALS	[35,36,37]
IGF-1	IGF-1R	HD; AD; PD; MND	[38,39,40,41]
IGF-2	IGF-2R/M6P receptor	HD	[42]
VEGF	VEGFR1/2; NRP1	AD; ALS; PD	[43,44,45]
FGF	FGFR1–4	AD; PD	[46,47]
CNTF	CNTFRα-gp130-LIFRβ	HD; ALS	[48]
NT-4/5	TrkB/p75NTR	PD	[49,50]
TGF-β	TGF-βRI/II	AD; PD; ALS	[51,52,53]
EGF	EGFR/ErbB	AD; PD	[54,55]

## 3. Modulating Aβ Clearance and Tau Hyperphosphorylation via the IGF-1/PI3K/Akt Axis in AD Pathogenesis

AD is an age-dependent neurodegenerative disorder characterized by progressive cognitive decline, and it is the primary cause of dementia [56]. Driven by global population aging, and as the leading cause of dementia, AD has become one of the most costly and burdensome diseases of this century. Dementia prevalence is projected to double in Europe and triple worldwide by 2050, and this estimate rises a further threefold when a biological rather than clinical definition of AD is applied [57]. The hallmark neuropathological changes of AD include three characteristics: extracellular Aβ deposition forming extracellular Aβ deposition forming neuritic (senile) plaques, intracellular tau protein overphosphorylation aggregation forming neurofibrillary tangles (NFT), and synaptic loss and cell death represented by cholinergic basal forebrain neurons [58,59,60]. At the Aβ metabolism level, amyloid precursor protein (APP) is sequentially hydrolyzed by β-secretase (BACE1) and γ-secretase to generate Aβ peptides, among which Aβ_42_, due to its aggregability, constitutes the core component of the plaque, surrounded by dystrophic neurites and activated glial cells [61,62]. At the tau pathology level, tau protein loses its ability to bind to microtubules due to abnormal phosphorylation, transforming from soluble microtubule-associated protein to insoluble aggregates, ultimately forming NFT [63]. Aβ and tau are not independently pathogenic. Aβ activates kinases such as GSK-3β to promote excessive tau phosphorylation, while tau deficiency alleviates Aβ-induced neurotoxicity [64,65]. These findings indicate that both proteins synergistically drive neuronal loss. Indeed, inhibiting abnormal tau aggregation improves cognition in AD mouse models, underscoring tau’s pivotal role downstream of Aβ [66].

IGF-1 serves as the primary ligand for IGF-1R, a transmembrane tyrosine kinase receptor. After IGF-1 binds to IGF-1R, it activates the receptor’s autophosphorylation, subsequently initiating downstream signaling pathways such as PI3K/Akt and Mitogen-activated protein kinase (MAPK), and mediating biological effects such as promoting cell survival, proliferation, and metabolic regulation. Converging evidence indicates that disruption of this axis is a hallmark of AD neuropathology. Alterations in insulin-related signaling and downstream PI3K/Akt activity have been described in AD-related research [67,68]. However, these findings do not by themselves establish an IGF-1R-specific causal mechanism underlying impaired Aβ clearance. This pathological condition is termed “type 3 diabetes” by some scholars, yet it remains undetermined whether cerebral insulin resistance is a primary driving factor or a secondary manifestation of neurodegeneration [69]. In experimental AD-related models, IGF-1 overexpression has been shown to reduce Aβ oligomer binding to hippocampal neurons, attenuate oxidative stress and dendritic spine loss, and prevent Aβ oligomer-induced memory impairment in mice [70]. However, these findings do not directly establish IGF-1R-mediated Aβ clearance through carrier-protein transport. To verify the impact of this molecular network on the progression of AD, researchers have constructed various animal models. For example, in APP/PS1 double transgenic mice, from mild cognitive impairment to advanced neurodegeneration at different disease stages, the relationship between IGF-1R signaling intensity and AD is nuanced, rather than a simple linear stage model [71]. The effects of IGF-1R signaling in AD appear context-dependent rather than uniformly beneficial or detrimental. Physiological activation of IGF-1R can suppress GSK-3β activity via the PI3K/Akt pathway, reduce excessive tau hyperphosphorylation, facilitate Aβ clearance, and thereby exert neuroprotective effects. However, constitutive IGF-1R activation in p44 transgenic models is associated with maladaptive receptor switching and amyloidogenic changes. These findings caution against assuming that simple enhancement or inhibition of IGF-1R signaling would be uniformly beneficial.

### 3.1. PTEN and SHIP Phosphatases in PIP3 Regulation: General Signaling Context and AD-Relevant Evidence

The phosphoinositide (PIPn) family constitutes a critical class of signaling lipids generated through the site-specific phosphorylation of phosphatidylinositol, governing essential cellular processes including signal transduction, membrane trafficking, and cytoskeletal dynamics [72]. Altered phosphoinositide signaling has been discussed in relation to microglial actin remodeling and phagocytic dysfunction in AD [73]. However, these pathway-level observations do not directly establish an IGF-1-dependent mechanism. Concurrently, dysregulated PI3K/Akt signaling in microglia skews them toward a persistent proinflammatory phenotype with impaired phagocytic clearance, thereby sustaining chronic neuroinflammation and accelerating neurodegeneration [74].

As a pivotal lipid second messenger, PIP3 functions primarily by recruiting proteins containing pleckstrin homology (PH) domains, such as Akt and PDK1, to the plasma membrane, thereby initiating downstream survival and metabolic cascades [75]. However, PIP3 signaling is not a unidirectional amplification process; its spatial and temporal dynamics are strictly constrained by negative feedback mechanisms mediated by specific lipid phosphatases [76]. Two primary classes of PIP3 phosphatases regulate these levels PTEN (phosphatase and tensin homolog) and SHIP (SH2-containing Inositol 5′-phosphatase) [77]. PTEN acts as a 3-phosphatase selectively removing the phosphate group at the D3 position of the inositol ring to convert PIP3 back into PIP2, effectively terminating the PI3K/Akt signal [78]. In contrast, SHIP functions as a 5-phosphatase, hydrolyzing the phosphate at the D5 position to generate PI (3,4)P2, a distinct signaling lipid that retains independent biological activities [79]. At the pathway level, SHIP1 membrane recruitment and activation are regulated through multiple molecular interactions [79]. These findings provide general biochemical context rather than direct evidence of IGF-1-dependent regulation in AD. This dual regulatory system ensures that PIP3 not only recruits signaling effectors but also engages mechanisms for its own termination, creating a sophisticated balance in cellular signaling [80]. The distinct phenotypic outcomes resulting from PTEN and SHIP mutations further underscore the unique and non-redundant roles of these enzymes and their respective lipid products in maintaining cellular homeostasis. It is worth emphasizing that the regulation of PIP3 by PTEN and SHIP is not redundant but differs fundamentally in nature [81]. PTEN removes the phosphate group at the 3-position to produce PIP2, which essentially terminates the signal, whereas SHIP dephosphorylates the 5-position to generate PI (3,4)P_2_, which functions as a signal converter [82]. The latter can independently activate PH domain-containing effector proteins such as TAPP1/2, and participate in processes including membrane ruffling and cell migration [78]. This distinction is genetically underscored by INPP5D, which encodes SHIP1 and has been identified as a late-onset AD risk locus in genome-wide association studies (rs35349669 and rs10933431; rs35349669 accounts for approximately 3.8% of AD genetic risk). INPP5D is expressed predominantly in microglia and inhibits TREM2–DAP12-dependent PI3K signaling, indicating that PIP3 dysregulation in AD arises from several non-redundant phosphatases operating in distinct cellular compartments rather than from a single enzyme. A second 5-phosphatase, SHIP2 (encoded by INPPL1), also hydrolyses PIP3 at the D5 position [83]; in AD brain, SHIP2 gene expression and protein solubility are altered in parallel with pathology progression, and SHIP2 accumulates in plaque-associated dystrophic neurites and astrocytes. However, INPPL1 variants show only nominal association with CSF p-tau and have not reached genome-wide significance, and these alterations remain correlative rather than causal. In addition, both PTEN and SHIP2 contain a C2 domain. Although both PTEN and SHIP2 contain C2 domains, their functions differ: the PTEN C2 domain contributes to membrane recruitment, whereas the SHIP2 C2 domain primarily modulates interdomain interactions rather than acting as a major membrane anchor [77]. Nevertheless, the PTEN- and SHIP-related mechanisms described above primarily represent general phosphoinositide biology and should not be interpreted as direct evidence that IGF-1 or IGF-1R manipulation regulates these processes in AD.

Against this framework, calorie restriction (CR) provides an important perspective on the dual properties of this axis. A fasting-mimicking dietary intervention has also been reported to reduce neuroinflammation and attenuate cognitive decline in AD models [84]. Caloric restriction has been shown to prevent the age-associated TrkA-to-p75NTR switch and to reduce downstream ceramide/BACE1/Aβ changes [85]. Potential intersections between caloric-restriction effects and IGF-1R signaling are discussed in Section 3.2 and remain inferential rather than established. These findings suggest a possible intersection between caloric restriction and IGF-1R signaling, but they do not establish a PTEN/SHIP2-mediated mechanism of caloric restriction.

### 3.2. IGF-1R Initiates the TrkA/p75NTR Phenotypic Transition via PI3K-Dependent Ceramide Activation

The role of IGF-1R signaling in basal forebrain cholinergic neurons is highly context-dependent. Physiological IGF-1R activation supports neuronal survival via PI3K/Akt mediated inhibition of GSK-3β and downstream anti-apoptotic effects. However, p44 transgenic mice exhibit enhanced IGF-1R signaling associated with an earlier TrkA-to-p75NTR receptor switch [86]. Thus, the effects of IGF-1R signaling may vary with experimental context, including signaling duration and disease stage.

**Figure 2:**
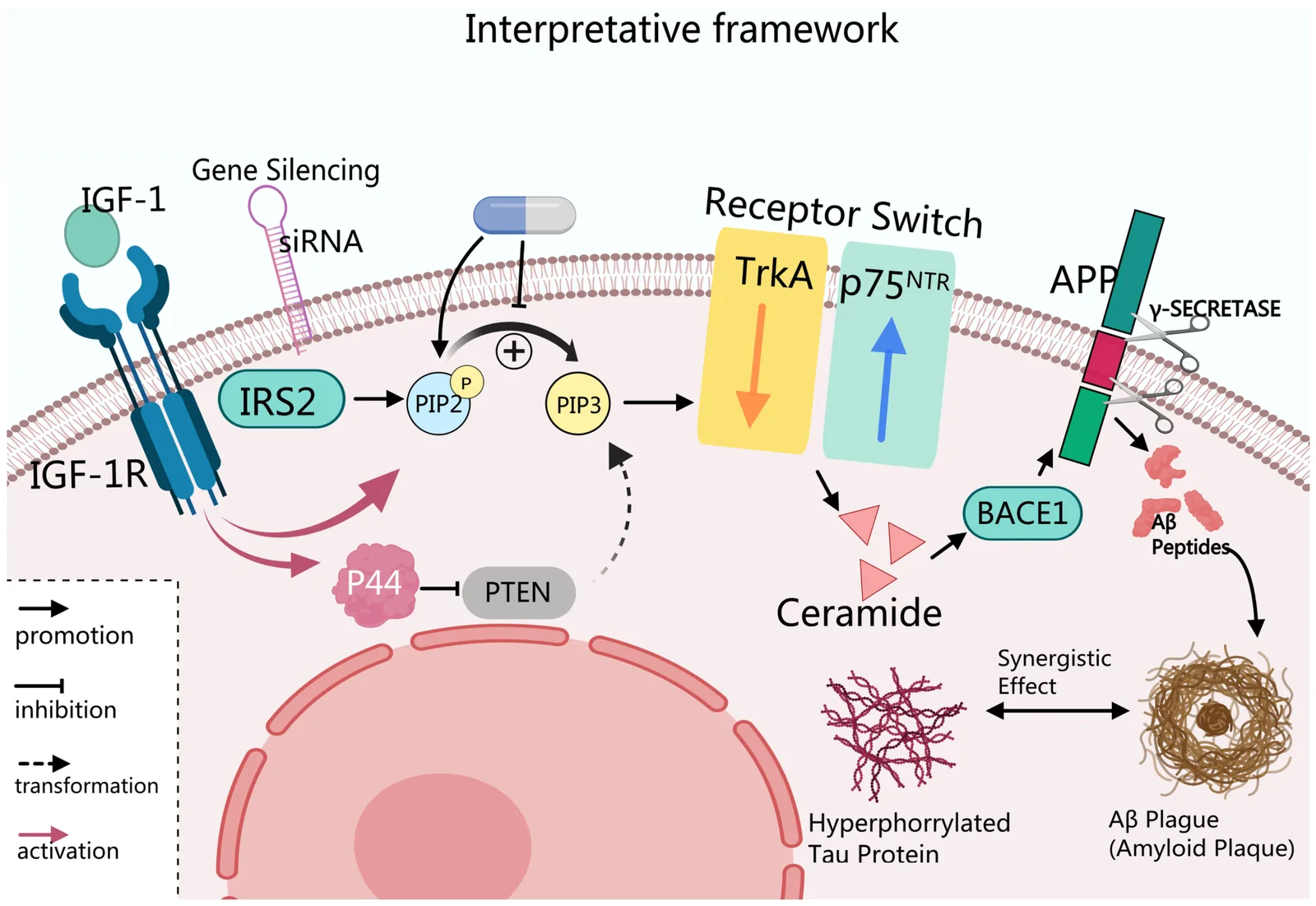
Interpretative framework of context-dependent IGF-1R signaling in AD pathogenesis. Created with MedPeer (https://www.medpeer.cn).

The phenotypic imbalance between TrkA and p75NTR on basal forebrain cholinergic neurons is a critical early event in AD pathology, precipitating amyloidogenesis and neuronal demise [87,88]. As illustrated in Figure 2, p75NTR upregulation activates ceramide, stabilizing BACE1 to drive Aβ production, while concurrent TrkA loss disinhibits GSK-3β, promoting tau hyperphosphorylation [85,87,88,89,90]. Thus, the TrkA/p75NTR switch converges on the dual pathological hallmarks of Aβ plaques and neurofibrillary tangles [91]. Experimental studies have implicated IGF-1R-dependent signaling in the regulation of TrkA and p75NTR expression [86,92], whereas broader interactions among neurotrophin receptors and PI3K/Akt signaling provide additional mechanistic context. These findings should not be generalized into a unified IGF-1-dependent regulatory mechanism across all AD models. The PI3K/Akt pathway serves as a central hub for neuronal survival, and its dysregulation leads to a cascade of pathological events including apoptosis, tauopathy, and synaptic loss [93]. Separately, impaired PI3K/Akt signaling may compromise neuronal survival and increase susceptibility to cellular stress [93,94,95]. However, these observations do not establish that reduced IGF-1R activity or decreased PIP3 production initiates the TrkA-to-p75NTR switch [86]. Furthermore, the upregulation of p75NTR not only promotes apoptosis directly but may also engage PIP3-dependent survival signaling bidirectionally—activating Akt via PI3K at low receptor levels while permitting JNK- and ceramide-driven death signaling to dominate at the higher levels attained in disease—rather than simply accelerating PIP3 depletion [96].

CR prevents the receptor phenotypic transformation of TrkA to p75NTR in mice, and restores the expression balance of TrkA/p75NTR. This effect prompts us to investigate whether IGF-1R is involved, as there are multiple mechanisms intersecting between the two. Studies conducted in human neuroblastoma cell lines and primary neurons have demonstrated that IGF-1R exerts regulatory effects upstream of TrkA and p75NTR [97]. Direct experimental studies indicate that the TrkA-to-p75NTR switch downstream of IGF-1R requires IRS2 and PI3K/PIP3/Akt signaling and is modulated by PTEN and p44. Hyperactivation of IGF-1R signaling in p44 transgenic mice was associated with an earlier receptor switch and increased Aβ production [86]. Gene silencing of IGF-1R or inhibition of its downstream PI3K can prevent the receptor phenotype transformation of TrkA to p75NTR. Notably, cultured primary neurons spontaneously undergo a receptor phenotypic transition in vitro resembling that observed in normal aging brains: early stages (0–3 days) are dominated by TrkA expression, whereas later stages (18–24 days) show dominance of p75NTR [86], indicating an intrinsic temporal programming of this transition. Once initiated, this transition triggers a downstream cascade: activation of the second messenger ceramide, enhanced BACE1 stability, and increased Aβ generation. Importantly, silencing IGF-1R gene expression blocks this entire cascade, confirming IGF-1R’s initiating role. In p44+/+ mice, which overexpress p44 and exhibit enhanced IGF-1R signaling, an earlier TrkA-to-p75NTR switch and increased Aβ generation have been observed [86]. Genetic and biochemical experiments further elucidate the molecular composition of this pathway: upon IGF-1R activation, the adaptor protein IRS2 recruits PI3K, catalyzing the conversion of PIP2 to PIP3 and sustaining pro-survival signaling downstream of TrkA.

PTEN negatively regulates the IGF-1R/IRS2/PI3K/PIP3-dependent receptor-switch pathway, whereas p44-associated IGF-1R hyperactivation accelerates the TrkA-to-p75NTR transition in the experimental models studied [86]. The “braking” function of PTEN within the “IGF-1R/PI3K/PIP3 switch” signaling axis aligns precisely with the previously described dynamic balance in PIP3 generation and loss.

In summary, IGF-1R/IRS2/PI3K/PIP3 signaling contributes to the TrkA-to-p75NTR switch in the experimental systems studied [86,92]. The extent to which this pathway mediates the CR-associated reversal of the receptor switch remains inferential and requires direct experimental validation.

### 3.3. PI3K/PIP3–Akt/GSK-3β Signaling and Tau Pathology: IGF-1-Related Evidence and Context

Throughout this review, descriptors of IGF-1R activation intensity (e.g., “transient/physiological”, “supraphysiological”, “chronic”, “reduced”) are anchored to the experimental conditions in which the corresponding effects were observed, rather than to a common quantitative scale; no study to date has mapped IGF-1R activation onto a shared dose axis across models. Accordingly, the context-dependent model summarized in Figure 2 is presented as an interpretative working hypothesis that organizes these heterogeneous findings, and not as a validated quantitative dose–response model.

Although caloric restriction has been reported to prevent the age-associated TrkA-to-p75NTR switch [85], it has not been established that this effect is directly mediated by reduced IGF-1R activity or PIP3 production. Moreover, reduced IGF-1R signaling did not protect against AD-related pathology in a recent Igf1r-haploinsufficient rat model [98]. These findings underscore the context dependence of IGF-1R signaling in NDDs. Physiological reduction of IGF-1R signaling can relieve metabolic burden and strengthen cellular stress resistance, yet the PI3K/Akt pathway is also critical for maintaining neuronal homeostasis, and its marked attenuation may promote oxidative stress, inflammatory responses and cell apoptosis [74]. Reduced PI3K/Akt signaling may relieve Akt-mediated inhibition of GSK-3β, a pathway-level mechanism that should be distinguished from the IGF-1R-dependent TrkA-to-p75NTR receptor switch. Ample evidence has demonstrated that overactivated GSK-3β promotes excessive phosphorylation of tau protein, leading to microtubule destabilization and neurodegeneration [99]. GSK-3β and tau protein form a key synergistic axis in the pathogenesis of AD [100]. Therefore, the pathological consequences caused by the weakened IGF-1R/PI3K/PIP3 signaling axis need to be interpreted from a broader perspective. Dysregulation of PI3K/PIP3/Akt signaling has been implicated in several AD-related pathological processes, including GSK-3β-associated tau pathology and disturbances in neuronal homeostasis [99,100,101]. However, these pathway-level associations do not establish that reduced PIP3 production initiates the TrkA-to-p75NTR receptor switch.

### 3.4. PIP2 Signaling and Neuronal Homeostasis: Potential Relevance to IGF-1R in AD

The dynamic balance between different types of phosphatidylinositol (PtdIns) is crucial for maintaining the normal physiological functions of cells, particularly in regulating membrane dynamics and signal transduction at the cytoplasmic membrane interface [72]. As a previously overlooked signaling molecule, the importance of PIP2 is gradually being recognized for its role beyond serving as an inert precursor for PIP3 production. The hydrolysis of PIP2 by phospholipase C (PLC) generates inositol 1,4,5-trisphosphate (IP3) and diacylglycerol (DAG), which participate in intracellular Ca2+ release and protein kinase C (PKC) activation. These reactions illustrate the broader functions of phosphoinositide metabolism but do not, by themselves, establish an IGF-1R-dependent mechanism in AD. The diverse functions of phosphoinositides provide important context for understanding neuronal signaling and metabolic regulation. However, the extent to which IGF-1R directly regulates these PIP2-dependent processes in AD remains uncertain. Therefore, the proposed relationship between IGF-1R signaling and PIP2-dependent neuronal homeostasis should be regarded as a mechanistic hypothesis rather than an established causal pathway.

Caloric restriction and fasting-mimicking dietary interventions have been associated with neuroprotective changes in experimental AD-related models [84,85]. Although PI3K/Akt–FoxO signaling is relevant to cellular stress responses, the studies discussed here do not directly establish that CR-mediated neuroprotection in AD depends on IGF-1R suppression or activation of a specific FoxO/SOD2/catalase pathway. PI3K/Akt signaling can regulate GSK-3β activity, and GSK-3β dysregulation has been implicated in tau pathology [99,100]. However, these pathway-level findings do not establish that altered IGF-1R activity directly causes tau pathology in AD. Separately, experimental studies have demonstrated that IGF-1R-dependent IRS2/PI3K/PIP3 signaling participates in the TrkA-to-p75NTR receptor switch, which is associated with downstream ceramide/BACE1/Aβ changes [86,92]. These findings do not support reduced IGF-1R signaling or decreased PIP3 production as an alternative trigger of the same receptor switch.

In conclusion, the IGF-1R/PI3K/Akt signaling pathway contributes to neuronal survival, whereas IGF-1R-dependent TrkA-to-p75NTR receptor switching has been demonstrated in specific experimental models [86,92]. These findings should be distinguished from the separate consequences of impaired PI3K/Akt signaling, including potentially increased GSK-3β activity and tau-related pathology [99,100]. The relationships among these mechanisms and AD progression require further investigation. Consequently, understanding the commonalities and differences in how various growth factors modulate neuronal survival requires an examination of their shared downstream effectors, specifically the phosphoinositide signaling molecules that govern membrane dynamics and signal transduction.

Addressing AD requires an interdisciplinary strategy integrating molecular biology, pharmacology, and clinical medicine. Additional investigation is required to elucidate the distinct contributions of PTEN and SHIP phosphatases to PIP3 regulation in AD [102]. Future pharmacological research could explore brain-penetrant modulators targeting these phosphatases or enhancing IGF-1R signaling, as potential strategies to mitigate neuronal loss and microglial-mediated neuroinflammation [103]. Future studies could investigate biomarker-stratified trial designs using INPPL1 expression or PIP3 metabolic profiles to identify responsive patients. Further validating IGF-1/IGF-1R modulation as a preventive strategy will depend on elucidating its interplay with GSK-3-driven tauopathy and amyloid-β metabolism. This approach addresses the multifactorial nature of AD and may contribute to a robust evidence base for multi-target therapeutic development that restores phosphoinositide homeostasis and reduces chronic inflammation.

## 4. Modulating α-Synuclein Homeostasis and Dopaminergic Neuroprotection via the IGF-1/PI3K/Akt Axis in PD Pathogenesis

**Figure 3:**
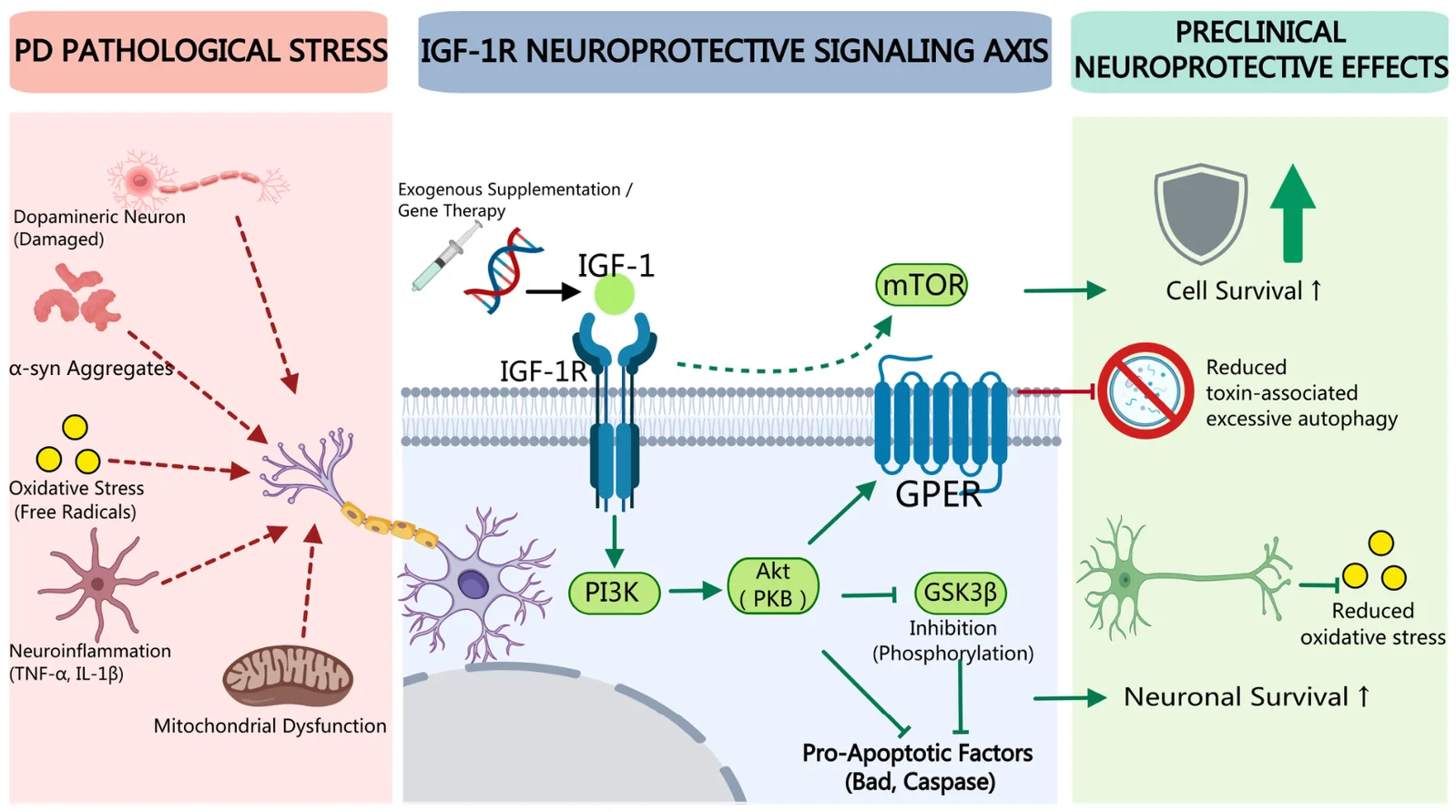
IGF-1-related neuroprotective signaling in preclinical PD models. Schematic showing IGF-1-related neuroprotective signaling in preclinical PD models, including inhibition of apoptosis and modulation of autophagy-related responses. Direct IGF-1-dependent CMA activation or α-synuclein clearance remains unestablished. Created with MedPeer (https://www.medpeer.cn).

PD is a common degenerative disease characterized by the progressive loss of dopaminergic neurons in the substantia nigra and abnormal aggregation of α-synuclein, forming Lewy bodies [104]. As the disease advances, α-synuclein pathology spreads to cortical and limbic regions, including the hippocampus, which is strongly associated with the development of dementia in PD [105]. The progressive degeneration of dopaminergic neurons within the substantia nigra pars compacta, coupled with the pathological accumulation of α-synuclein aggregates known as Lewy bodies, constitutes the primary neuropathological hallmark of PD [106]. As illustrated in Figure 3 (left panel), this degeneration is driven by multiple converging pathological stresses: α-synuclein aggregation, oxidative stress, neuroinflammation, and mitochondrial dysfunction, all of which converge to impair neuronal viability and disrupt basal ganglia circuitry. These pathological changes disrupt the basal ganglia circuitry, leading to the cardinal motor symptoms of bradykinesia, rigidity, and tremor. As the disease progresses, patients often experience worsening motor dysfunction, including gait disturbances and speech impairments, which significantly impact quality of life [107]. Cognitive impairment is a prevalent non-motor symptom that constitutes a vital aspect of PD progression [108]. Studies indicate that a significant proportion of PD patients develop mild cognitive impairment (MCI), and approximately 20% may progress to Parkinson’s disease dementia (PDD) within three years of MCI diagnosis [109,110]. Longitudinal data suggests that up to 70% of PD patients may develop dementia after eight years of disease duration [111]. PDD is clinically characterized by executive dysfunction, memory deficits, and cognitive fluctuation [112]. Notably, while some non-motor symptoms precede motor onset, significant cognitive decline typically occurs after the establishment of motor symptoms in classic PD, distinguishing it from dementia with Lewy bodies where cognitive issues arise earlier [113]. Although there is no cure for PD, early diagnosis and standardized management can alleviate symptoms and improve patient outcomes [114].

In the pathological context of PD, IGF-1 mediates comprehensive neuroprotective actions via activation of the IGF-1R/PI3K/Akt axis. The left panel (Figure 3) presents the core pathologies of PD (dopaminergic neuron injury, α-synuclein aggregation, oxidative stress, inflammation, and mitochondrial dysfunction). The middle panel shows that after IGF-1 activates IGF-1R, it inhibits GSK-3β (reducing pro-apoptotic factors) and regulates mTOR (suppressing excessive autophagy) through the PI3K/Akt pathway, and functions synergistically with GPER. The right panel summarizes neuroprotective outcomes reported in preclinical PD models. This pathway represents one mechanistic framework through which IGF-1 may exert neuroprotective effects in preclinical PD models. Emerging evidence suggests that serum IGF-1 levels may serve as a potential biomarker for PD, with studies reporting correlations between IGF-1 levels and disease severity or cognitive status [115]. In a two-year longitudinal study of patients with early, drug-naïve PD, baseline serum IGF-1 levels were associated with subsequent cognitive performance [109]. However, diagnosis relying on a single biomarker is insufficient. A multi-dimensional approach combining α-synuclein detection, olfactory testing, and imaging biomarkers is required for accurate prodromal screening and disease staging [116]. The neuroprotective potential of IGF-1 is mediated through the IGF-1R/PI3K/Akt signaling pathway, which inhibits apoptotic cascades and reduces dopaminergic neuron death [117]. Additionally, IGF-1 signaling may modulate toxin-associated autophagy via the Akt/mTOR axis [118]. Although IGF-1 may interact with other receptors such as the G protein-coupled estrogen receptor (GPER), preclinical evidence supports IGF-1R/PI3K/Akt signaling as one pathway through which IGF-1 may support dopaminergic neuron survival; disease-modifying effects in patients have not been established.

### 4.1. IGF-1-Associated PI3K/Akt Signaling and Phosphoinositide Context in PD

The following discussion distinguishes direct IGF-1 intervention studies involving PI3K/Akt signaling in PD-related models [117,118] from broader evidence concerning phosphoinositide regulation and α-synuclein pathology. IGF-1 has shown neuroprotective effects in cellular and animal PD models through IGF-1R/PI3K/Akt signaling [117]. This pathway supports neuronal survival in preclinical PD models; however, direct IGF-1-dependent effects on α-synuclein aggregation remain incompletely established [119]. The α-synuclein is a key structural protein of the presynaptic membrane under normal physiological conditions and participates in synaptic plasticity regulation, membrane structure maintenance, and neurotransmitter release. Under pathological conditions, α-synuclein undergoes conformational changes and is marked by abnormal phosphorylation at sites such as Ser129, forming insoluble aggregates, especially significantly deposited in dopaminergic neurons [120,121]. Notably, dopamine itself can produce reactive quinones through oxidative metabolism and covalently bind with α-synuclein, inhibiting its normal folding and degradation, thereby forming a malignant self-amplifying loop of dopamine oxidation and α-synuclein aggregation. IGF-1/PI3K/Akt signaling may support neuronal resilience in this pathological context. When α-synuclein accumulates abnormally in dopaminergic neurons, its interaction with the Akt signaling pathway shows that it is context-dependent rather than unidirectional and it is highly dependent on the pathological stage [122]. On the one hand, α-synuclein oligomers can indirectly inhibit the activation of the PI3K/Akt pathway, downregulate the phosphorylation level of Akt at the Ser473 site, and thereby relieve the inhibition of pro-apoptotic factors Bad and FoxO on Akt, reducing the neuronal survival ability. The mechanism mainly involves the binding of α-synuclein oligomers to the TrkB kinase domain, blocking the BDNF/TrkB signal to the PI3K, causing Akt to be insufficiently phosphorylated due to the loss of upstream driving [123]. At the same time, however, α-synuclein overexpression drives mTORC1/S6K1-dependent inhibitory phosphorylation of IRS-1 at Ser636 [122]; initiating negative feedback regulation, ultimately resulting in the gradual weakening of upstream signals in the PI3K/Akt pathway and the progressive attenuation of neuroprotective signals [124].

In summary, direct preclinical evidence supports a role for IGF-1/IGF-1R signaling in promoting PI3K/Akt-dependent neuronal survival in PD models. In contrast, the relationships among α-synuclein, TrkB, mTORC1/S6K1, and GSK-3β are supported largely by experiments that did not manipulate IGF-1. These findings provide mechanistic context but do not by themselves demonstrate that IGF-1 directly modifies α-synuclein aggregation or clearance.

### 4.2. GSK-3β and α-Synuclein Pathology in PD: Pathway-Level Evidence

The PI3K/Akt pathway can regulate GSK-3β through Akt-mediated phosphorylation at Ser9 [125,126]. Although IGF-1 can activate PI3K/Akt signaling in experimental systems, this general pathway relationship does not establish that IGF-1 directly regulates α-synuclein Ser129 phosphorylation in PD. GSK-3β is a constitutively activated serine/threonine protein kinase that maintains basal kinase activity in the absence of upstream inhibitory signals. GSK-3β is widely expressed in neurons and participates in regulating key life processes such as cell growth, differentiation, and apoptosis. Abnormal activity of GSK-3β is an important trigger for various NDDs [127]. GSK-3β is one of the key kinases that mediate the excessive phosphorylation of α-synuclein at the Ser129 site [128]. Abnormal phosphorylation of this site enhances the aggregation ability of α-synuclein, promoting its transformation from soluble monomers to insoluble fibers, and ultimately accelerating the formation of Lewy bodies.

GSK-3β has been identified as one of the kinases capable of phosphorylating α-synuclein at Ser129. This site is a neuropathological hallmark of Lewy body disease [129]. At the same time, the continuous activation of GSK-3β also aggravates oxidative stress and mitochondrial damage, creating a more favorable intracellular microenvironment for α-synuclein misfolding. In a 6-OHDA rat model, IGF-1 treatment was associated with increased Akt phosphorylation, inhibition of GSK-3β-related pro-death signaling, and protection of nigrostriatal dopaminergic neurons [130]. Separately, GSK-3β dysregulation has been linked to α-synuclein and tau pathology in PD models [128,129]. Because these observations derive from different experimental systems, they support pathway-level plausibility but do not establish that IGF-1 directly reduces α-synuclein Ser129 phosphorylation.

Thus, IGF-1/IGF-1R signaling can modulate Akt/GSK-3β- and mTOR-related pathways in experimental PD models, whereas direct evidence that IGF-1 enhances autophagic flux, activates CMA, or promotes α-synuclein clearance remains insufficient.

### 4.3. IGF-1 and Autophagy-Related Processes in PD

Autophagy-related evidence directly involving IGF-1 in PD is currently limited. In MPTP-treated mice and MPP+-treated SH-SY5Y cells, IGF-1 pretreatment reversed toxin-associated increases in LC3-II and decreases in p62 and restored Akt/mTOR phosphorylation; these effects were attenuated by IGF-1R or GPER antagonism [118]. These findings support inhibition of toxin-associated excessive autophagy under the experimental conditions tested, rather than enhancement of autophagic flux.

CMA dysfunction, LAMP-2A regulation, impaired lysosomal degradation, and α-synuclein accumulation are independently relevant to PD pathogenesis. However, the studies cited for these processes largely do not directly manipulate IGF-1 or IGF-1R. Therefore, a direct IGF-1→CMA→α-synuclein-clearance pathway cannot currently be concluded from these data. Such relationships are considered here as mechanistic hypotheses requiring direct experimental validation.

In summary, direct preclinical studies support neuroprotective effects of IGF-1 through PI3K/Akt-associated signaling in selected PD-related toxin models [117,118,130]. In contrast, evidence concerning α-synuclein homeostasis, CMA, and broader autophagy regulation should be distinguished from these direct IGF-1 intervention findings. Targeting this pathway remains a preclinical strategy of interest in PD, although disease-modifying efficacy in patients has not been established. As pathway-level context, autophagy dysfunction is broadly implicated in neurodegenerative disease, and AMPK–mTOR–ULK signaling contributes to the regulation of autophagy initiation [131,132].

## 5. IGF-1 Signaling and Iba-1-Related Neuroinflammatory Context in MND Pathogenesis

MND is a progressive NDD that commonly occurs in middle-aged and elderly people [133]. Clinically, patients develop progressive, painless muscle weakness and wasting—often beginning focally in the limbs or bulbar muscles—with ALS, in which upper and lower motor neuron signs coexist, being the most common phenotype, whereas pure lower or upper motor neuron variants correspond to progressive muscular atrophy and primary lateral sclerosis, respectively [134]. Its clinical manifestations include weakness or paralysis of the upper and lower limbs, increased muscle tension, hyperactive tendon reflexes, positive pathological reflexes and other symptoms. Due to the lack of specific therapeutic approaches, early diagnosis and early treatment of this disease are of great importance [135,136].

In the ALS Registry Swabia study, serum IGF-1 concentrations were not significantly associated with ALS risk, whereas higher IGF-1 concentrations were associated with a more favorable survival prognosis among patients with ALS [137].

Recent studies have revealed the pivotal role of Iba-1 in the pathological processes of neuroinflammation and MND. Iba-1 (Ionized calcium-binding adapter molecule 1) is a specific marker protein for microglia and macrophages. It mainly exerts vital functions in the innate immune responses of the central microglia and peripheral macrophages by regulating the activation state, morphological changes of microglia and the release of inflammatory factors [138]. Under normal physiological conditions, Iba-1 helps maintain the homeostasis of microglia, while in NDDs, its abnormal expression level is often closely correlated with the severity of the diseases [139].

Studies have shown that IGF-1 exerts a protective effect on skeletal muscle cells [140]. Preclinical studies have demonstrated protective effects of IGF-1 in skeletal muscle cell models [141,142]. Separately, muscle-specific IGF-1 expression has been shown to protect motor neurons and delay disease progression in a SOD1G93A mouse model of ALS [143]. By regulating neuron apoptosis-related genes, IGF-1 can protect skeletal muscle cells from oxidative stress damage [141,142]. Dobrowolny et al. demonstrated that local IGF-1 overexpression in skeletal muscle protects motor neurons and extends survival in SOD1G93A mice, supporting a trophic effect of IGF-1 in this preclinical ALS model [143]. However, the precise mechanisms by which IGF-1 signaling is modulated in MND skeletal muscle remain incompletely understood.

## 6. Modulating Mutant Huntingtin Toxicity and Mitochondrial Dysfunction via the IGF-1/Akt Axis in HD

HD is an autosomal dominant neurodegenerative disorder caused by the expansion of CAG repeat sequences in the first exon of the HTT gene. This mutation leads to the production of mutant huntingtin protein (mHTT). Its clinical manifestations mainly affect three major aspects: motor function, cognition and mental state.

In the early stage of the disease, enkephalin-expressing indirect pathway neurons projecting to the external globus pallidus selectively degenerate preferentially [23], which is equivalent to removing the “brake” function in the basal ganglia motor circuit, leading to thalamic disinhibition and chorea. As the disease progresses, direct pathway neurons are also gradually involved, and the motor symptoms shift from predominant choreiform movements to Parkinson-like manifestations dominated by rigidity and bradykinesia [23]. Juvenile HD particularly often onsets with rigidity and gait disorders. Cognitive impairment usually occurs prior to or concurrently with motor symptoms, mainly manifested as declines in executive function, attention and other abilities. Psychiatric symptoms may appear earlier than motor and cognitive symptoms, mainly including depression (with an incidence rate of over 40%), irritability and aggressiveness, apathy, anxiety and compulsive behaviors. Although there is still no radical cure for HD at present, and clinical management focuses on multidisciplinary collaborative symptom control [144], studies have shown that circulating IGF-1 can be used to predict early clinical cognitive decline [17].

The IGF-1/Akt pathway mediates the phosphorylation of mHTT Ser421 by activating Akt, which directly reduces the toxicity of mutant proteins and inhibits the formation of intranuclear inclusions. Activated Akt phosphorylates arfaptin 2 at Ser260 and rescues proteasome function, whereas restoration of IGF-1 signaling attenuates aberrant caspase-6 activation and reduces the toxic N-terminal 586 fragment [145]; together with Akt-mediated huntingtin Ser421 phosphorylation [146], these actions counteract mHTT-induced neuronal death through multiple mechanisms. In addition, the IGF-1/Akt signal can remarkably ameliorate HD-related mitochondrial dysfunction, reduce reactive oxygen species production and restore energy metabolism homeostasis, serving as a crucial metabolic foundation for its neuroprotective effects. Collectively, these findings establish IGF-1/Akt signaling as a convergent nodal point that counteracts HD pathogenesis through coordinated actions: directly blunting mHTT toxicity via Ser421 phosphorylation, reinstating proteasomal degradation through arfaptin 2, and correcting mitochondrial bioenergetic deficits, thereby extending the neuroprotective repertoire of the IGF-1R/PI3K/Akt axis across distinct neurodegenerative contexts.

Animal experiments have confirmed that IGF-1 can effectively protect striatal neurons and ameliorate metabolic abnormalities in HD models dominated by excitotoxicity [29]. Intranasal delivery has improved disease-related phenotypes in model animals, supporting further preclinical evaluation of this delivery approach. However, the protective effects of this pathway are distinctly condition-dependent, and it may even exacerbate damage in pathological models with severe mitochondrial inhibition, indicating that its functions are associated with pathological processes relevant to HD [147,148,149].

The paradox of elevated peripheral IGF-1 levels accompanied by accelerated cognitive decline at the clinical level reflects complex relationships such as uncoupling of central and peripheral signals, compensatory upregulation, and bidirectional regulation between HTT and IGF-1 (Table 2), and also poses significant challenges for clinical translation [150]. Meanwhile, the cross-regulation between manganese ions and the IGF-1/Akt pathway offers new insights for trace element intervention. Accordingly, the IGF-1/Akt pathway links protein toxicity, mitochondrial function and cell survival, providing potential therapeutic targets for HD.

**Table 2:** Advances in IGF-1 research in HD.

Theme	Core Finding	Model/Evidence	Significance
Akt-Mediated mHTT Phosphorylation	IGF-1 activates Akt to phosphorylate mutant Huntingtin (mHTT) at Ser421, reducing nuclear aggregation and neuronal death [151].	In vitro cell culture; human brain tissue; in vivo rat models.	Establishes the first direct molecular link between growth factor signaling and mHTT toxicity.
Context-Dependency of Protection	IGF-1 protects against excitotoxicity [147] but exacerbates 3-NP-induced neurotoxicity [149].	In vivo rat models (Quinolinic Acid vs. 3-NP).	Demonstrates that IGF-1 is not a universal protectant; efficacy is strictly dependent on the underlying pathological driver.
Mitochondrial Rescue	IGF-1/Akt restores mitochondrial dynamics, reduces ROS [152], and improves ATP synthesis and Ca^2+^ buffering [153].	HD knock-in striatal cells; patient-derived lymphoblasts.	Supports a role for mitochondrial dysfunction in IGF-1-related neuroprotective effects.
The Clinical Paradox	Elevated plasma IGF-1 levels in HD patients predict faster cognitive decline [150].	Prospective cohorts (n > 150).	Suggests that CNS bioavailability may be more informative than systemic concentration for future therapeutic evaluation.
Bidirectional Regulation	Full-length HTT regulates IGF-1 transcription; loss of HTT function leads to IGF-1 deficiency [41].	Multi-strain transgenic mice (YAC/BACHD).	Reveals a feedback loop between HTT and IGF-1, supporting further investigation of IGF-1-related interventions.
Intranasal Delivery Breakthrough	Intranasal IGF-1 targets the brain, restoring p-Akt and motor/metabolic phenotypes without elevating plasma IGF-1 [148].	YAC128 mouse model.	Provides preclinical evidence that intranasal delivery can enhance CNS exposure while limiting peripheral exposure.
IGF-2 Complementarity	IGF-2 reduces mHTT aggregates via AKT and NF-κB signaling [154].	In vivo and in vitro HD models.	Supports further investigation of IGF-2 as a potentially complementary IGF-family pathway.

## 7. Literature Search Strategy

This article is a narrative review rather than a systematic review. To improve the reproducibility and transparency of the literature-selection process, the revised manuscript now reports the detailed PubMed search strategy, search fields, filters, search date, screening flow, and reference-level list of cited literature.

The literature search was conducted in PubMed. For the reproducibility audit conducted during this revision, the PubMed searches were rerun on 20 September 2026, with publication dates restricted to 1 January 2015 through 6 September 2026. The exact database-specific search strings are provided in Supplementary Table S1. Searches were restricted to English-language publications dated from 1 January 2015 to 6 September 2026; no additional article-type filter was applied at the database-search stage. Pre-2015 references cited for foundational context were identified separately and were not counted as products of the 2015–2026 database searches. The search results were screened according to predefined eligibility criteria, which included English-language original research articles and reviews addressing IGF-1/IGF-1R signaling, mechanistic processes, biomarkers, or therapeutic strategies relevant to AD, PD, MND/ALS, HD, or neurodegeneration more broadly.

Disease-specific yields were: Alzheimer’s disease, 304 records; Parkinson’s disease, 109; motor neuron disease/amyotrophic lateral sclerosis, 50; Huntington’s disease, 24; and the broader neurodegenerative-disease search, 410 records. Because many disease-specific reports do not carry the umbrella term “neurodegenerative diseases”, the disease-specific and umbrella searches are complementary rather than nested, and the yields are reported separately rather than summed. This distribution accounts for the differing depth of coverage accorded to each disorder in Section 3, Section 4, Section 5 and Section 6. The yields from each individual search, including the supplementary searches, are reported separately in Supplementary Table S1.

Records from the primary and supplementary searches were pooled and deduplicated using PubMed identifiers (PMIDs). Across the 13 PubMed searches, 3083 records were retrieved before deduplication. After removal of 622 duplicate records, 2461 unique records remained for title/abstract screening. Of these, 2218 records were excluded during title/abstract screening, and 243 articles underwent full-text assessment. Following full-text assessment, 84 articles were excluded for the following primary reasons: outside the predefined disease or topic scope (n = 18), insufficient direct relevance to IGF-1/IGF-1R signaling (n = 30), lack of relevant mechanistic, biomarker, or translational information (n = 29), and ineligible publication types (n = 7). Each excluded article was assigned one primary exclusion reason to avoid double counting. A total of 159 articles were retained following full-text screening. The narrative synthesis included both primary research articles and review articles. Primary studies provided original experimental or clinical findings, whereas reviews contributed broader background information and secondary synthesis. The first and corresponding authors independently screened the titles and abstracts and subsequently assessed the full texts of potentially eligible articles. Disagreements regarding eligibility were resolved through discussion until consensus was reached.

In addition, a secondary search was conducted using the terms “IGF-1” and “PI3K/Akt” or “IGF-1” and “neuroinflammation” or “IGF-1” and “autophagy” or “IGF-1” and “blood-brain barrier” or “IGF-1” and “PTEN” or “IGF-1” and “SHIP2” or “IGF-1” and “intranasal delivery” or “IGF-1” and “nanocarrier.” Supplementary searches used the same publication-date and language restrictions as the primary searches. The results were screened based on the inclusion criteria: English articles focusing on mechanistic insights, translational strategies, or therapeutic interventions involving IGF-1. Studies meeting the above criteria were included after screening the titles, abstracts, and full texts. A reference-level dataset of the literature contributing to the narrative synthesis, including citation details, review section/topic, and inclusion basis, is provided in Supplementary Table S2. The exact supplementary-search strings and the number of records retrieved from each query are provided in Supplementary Table S1.

For transparency regarding inferential strength, the studies contributing to Section 3, Section 4, Section 5 and Section 6 are grouped according to evidence source and study design: human interventional studies, human observational or biomarker studies, post-mortem tissue or patient-derived studies, in vivo animal studies, and in vitro studies. These categories describe the source and design of the evidence and should not be interpreted as a formal ranking of methodological quality or risk of bias. Accordingly, Table 3 has been reorganized as a study-level evidence map. Each row now presents a specific study or closely related experimental dataset, links the reported finding directly to its supporting reference, and identifies the principal study-specific limitation or interpretive constraint. Human interventional findings are distinguished from observational associations and preclinical mechanistic evidence.

**Table 3:** Study-level evidence map of IGF-1 research across major neurodegenerative diseases.

Disease	Evidence Source ^1^	Study Design and Model	Key Finding (Reference)	Effect ^2^	Study-Specific Limitation/Interpretive Note
AD	C	Post-mortem hippocampal tissue from patients with AD	The active/inactive IGF-1 ratio was reduced, whereas IGF-1R expression was increased, in AD hippocampal samples [70].	↓	Observational human-tissue dataset; cannot determine whether altered signaling is causal or secondary.
AD	D	Aging mouse models with caloric restriction	Caloric restriction prevented the age-associated TrkA-to-p75NTR receptor switch and associated Aβ-generating changes [85].	↑	Aging-model intervention; a direct IGF-1R-mediated effect of caloric restriction was not demonstrated.
AD	D	p44 transgenic mice exhibiting enhanced IGF-1R signaling	Enhanced IGF-1R signaling was associated with an earlier TrkA-to-p75NTR switch and increased brain Aβ production [86].	↓	Specific accelerated-aging transgenic model; findings cannot be generalized to all IGF-1R activation states or human AD.
AD	D	Mouse hippocampi exposed to Aβ oligomers; IGF-1R inhibition	Aβ oligomers increased hippocampal IGF-1R phosphorylation and p75NTR expression; IGF-1R inhibition prevented the p75NTR increase [97].	↓	Acute experimental exposure model; does not establish the same mechanism across human AD stages.
PD	B	Cross-sectional PD patient and healthy-control cohorts	Serum IGF-1 levels were associated with anxiety, depression, and cognitive measures in patients with PD [115].	~	Cross-sectional association; peripheral IGF-1 does not directly measure CNS signaling or establish causality.
PD	B	Early, drug-naïve PD cohort; 2-year follow-up	Lower baseline serum IGF-1 was associated with poorer performance or faster decline on selected cognitive tests over two years [109].	~	Small observational longitudinal cohort; predictive associations are not evidence of a treatment effect.
PD	E	MPP^+^-exposed SH-SY5Y cells treated with IGF-1	IGF-1 reduced MPP^+^-induced apoptosis and oxidative injury through PI3K/PDK1/Akt-associated signaling [117].	↑	In vitro toxin-based cell line model; no in vivo or clinical therapeutic efficacy established.
PD	D	MPTP-treated mice receiving IGF-1	IGF-1 pretreatment attenuated toxin-associated dopaminergic injury and changes in LC3-II and p62; IGF-1R or GPER antagonism attenuated these effects [118].	↑	Acute toxin-based mouse model; autophagy-marker changes alone do not prove altered autophagic flux.
MND/ALS	B	ALS Registry Swabia case–control and prognostic cohort	Serum IGF-1 was not significantly associated with ALS risk after adjustment; higher concentrations were nonlinearly associated with lower mortality among ALS cases [137].	~	Observational peripheral biomarker evidence; nonlinear association and residual confounding limit causal interpretation.
MND/ALS	D	SOD1G93A mice with skeletal-muscle-restricted mIGF-1 expression	Muscle-restricted local IGF-1 expression preserved neuromuscular junctions and motor neurons and prolonged survival in SOD1G93A mice [143].	↑	Single transgenic ALS model; muscle-targeted genetic expression is not equivalent to systemic IGF-1 treatment.
MND/ALS	D	SOD1G93A mice receiving an IGF-1R inhibitor	IGF-1R inhibition increased retrograde signaling-endosome transport velocity in motor axons of SOD1G93A mice [40].	↑	Acute pharmacologic inhibition improved a transport endpoint; survival benefit and effects of chronic inhibition were not tested.
HD	B	Longitudinal HD patient cohort; circulating IGF-1	Higher circulating IGF-1 levels were associated with worse cognitive performance and cognitive decline over follow-up [150].	~	Observational biomarker evidence; the association does not establish IGF-1 as a cause of cognitive deterioration.
HD	E	Cultured neuronal models expressing mutant huntingtin; IGF-1/Akt intervention	IGF-1-activated Akt phosphorylated huntingtin and reduced mutant huntingtin-induced neuronal death and intranuclear inclusions [146].	↑	Mechanistic cell-based study; relevance to clinical IGF-1 treatment has not been established.
HD	D	BACHD transgenic mice carrying S421 phosphomimetic or phosphoresistant mHTT variants	Phosphomimetic S421 mutation attenuated mHTT-related behavioral dysfunction and striatal degeneration and promoted mHTT turnover [151].	↑	Genetic phosphomimetic manipulation, not direct IGF-1 treatment; therapeutic transferability remains unknown.
HD	D	YAC128 HD mice receiving intranasal recombinant human IGF-1	Intranasal IGF-1 improved motor activity, metabolic abnormalities, and Akt/mHTT-Ser421 phosphorylation-related measures in YAC128 mice [148].	↑	Two-week intervention in one HD mouse model; human delivery, safety, and efficacy are unestablished.
HD	D	Rats receiving 3-nitropropionic acid (3-NP) and intracerebral IGF-1	Higher-dose IGF-1 infusion exacerbated 3-NP-induced striatal neurotoxicity, whereas lower-dose infusion did not provide protection [149].	↓	Mitochondrial toxin model with dose-dependent results; cannot be generalized to all HD models or IGF-1 regimens.

^1^ Evidence-source categories: A = human interventional studies; B = human observational or biomarker studies; C = post-mortem human tissue or patient-derived studies; D = in vivo animal studies; E = in vitro studies. These categories describe study design and evidence source and do not constitute a formal ranking of methodological quality or risk of bias. ^2^ Effect symbols: ↑ protective/beneficial or signal restored; ↓ harmful, signaling loss, or adverse prognostic association; ↔ context-dependent or directionally opposing findings; ↑/↓ direction depends on receptor compartment; ~ association only. Symbols summarize the direction reported in the source study, not a clinical recommendation. Effect symbols across evidence-source categories denote associations or preclinical effects and should not be interpreted as treatment effects demonstrated in patients.

## 8. Concluding Remarks

With the development of molecular biology and neuropharmacology, IGF-1 is a neurotrophic factor with neuroprotective effects demonstrated primarily in cellular and animal models, motivating continued mechanistic and translational investigation in NDDs. Although research reports on the IGF-1 signaling pathway have been increasing year by year, most of the research achievements remain in the preclinical research stage, and efficacy evaluations are mostly limited to animal models. Clinical efficacy has not yet been established, and further translational validation is required. IGF-1-based approaches, including recombinant protein delivery and gene therapy, face several translational challenges, such as brain delivery, optimal intervention timing, and long-term safety. Although intranasal and viral-vector delivery have shown potential in preclinical models, specific therapeutic windows and safety-monitoring strategies have not been established for IGF-1-based treatment of NDDs in humans. Future studies could investigate the optimal timing of intervention and evaluate potential systemic adverse effects, including disturbances in glucose homeostasis and other long-term safety outcomes. Future studies could investigate how IGF-1-related signaling interacts with neuroinflammation, metabolic homeostasis, autophagy-related processes, and synaptic plasticity in disease-specific experimental models, and assess the potential benefits of targeting these pathways. IGF-1-based neuroprotective strategies therefore remain investigational, and their clinical utility requires validation in appropriately designed human studies.

This review summarizes the reported roles and mechanisms of IGF-1 across major NDDs. Innovatively, it conducts parallel comparison and sorting of its differentiated pathological mechanisms, signaling pathways and therapeutic strategies based on disease types, providing perspectives for future mechanistic and translational research on the IGF-1 signaling pathway.

Currently, the clinical translation of IGF-1-targeting approaches faces several challenges, including limited brain delivery, uncertainty regarding the optimal timing of intervention, and potential systemic adverse effects. Most of the evidence included in this review is derived from preclinical animal models. Such models fail to fully replicate the prolonged disease course and complex pathological mechanisms of human disorders. Consequently, promising findings obtained from animal studies often fail to be validated in human clinical trials, highlighting the substantial translational gap between laboratory research and clinical practice.

Potential biomarker-related directions for future IGF-1 research. Heterogeneity in IGF-1-related signaling across diseases, experimental models, and patient populations warrants further investigation. Whether serum or CSF IGF-1 levels, IGFBP profiles, or IGF-1R phosphorylation status can inform patient stratification or dosing strategies in clinical trials remains to be established. Future studies could evaluate biomarker stratification, context-dependent dosing strategies, and combination approaches. The p44/PTEN findings in AD and analogous context-dependent observations in PD should be regarded as preclinical hypotheses rather than validated clinical dosing frameworks. Biomarker-guided and context-specific IGF-1 modulation may provide a framework for future preclinical and clinical investigation, but its therapeutic benefit remains to be established.

This review summarizes recent progress and future research directions in IGF-1 signaling across NDDs and may inform future translational studies evaluating the clinical relevance of IGF-1-related interventions.

## Data Availability

No new data were created or analyzed in this study. Data sharing is not applicable to this article.

## Supplementary Materials

The following supporting information can be downloaded at: https://www.mdpi.com/article/10.3390/ijms27219696/s1.

## Abbreviations

IGF-1	Insulin-like growth factor-1
AD	Alzheimer’s disease
PD	Parkinson’s disease
MND	Motor neuron disease
ALS	Amyotrophic lateral sclerosis
HD	Huntington’s disease
RD	Retinal Degeneration
SCI	Spinal Cord Injury
PN	Peripheral Neuropathy
Aβ	Amyloid β-peptide
NFT	Neurofibrillary tangles
APP	Amyloid precursor protein
IGF-1R	Insulin-like growth factor 1 receptor
PI3K	Phosphatidylinositol 3-kinase
Akt/PKB	Protein kinase B
mTOR	Mammalian target of rapamycin
GSK-3β	Glycogen synthase kinase 3β
MAPK/ERK	Mitogen-activated protein kinase/extracellular signal-regulated kinase
CREB	cAMP-response element-binding protein
PTEN	Phosphatase and tensin homolog
PIP2	Phosphatidylinositol 4,5-bisphosphate
PIP3	Phosphatidylinositol 3,4,5-trisphosphate
IRS2	Insulin receptor substrate 2
BACE1	β-site APP cleaving enzyme 1
PDD	Parkinson’s disease dementia
Iba-1	Ionized calcium-binding adaptor molecule 1
TNF-α	Tumor necrosis factor-α
IL-6	Interleukin-6
NGF	Nerve growth factor
TrkA	Tropomyosin receptor kinase A
p75NTR	p75 neurotrophin receptor
GPER	G protein-coupled estrogen receptor
MPP^+^	1-methyl-4-phenylpyridinium
6-OHDA	6-hydroxydopamine
MPTP	1-methyl-4-phenyl-1,2,3,6-tetrahydropyridine
PFF	Preformed fibril
siRNA	Small interfering RNA
HGH	Human growth hormone
CNKI	China National Knowledge Infrastructure
Scopus	Scopus database
Da	Dalton
