# Supplementary material for: Research Progress of Insulin-like Growth Factor-1 in Major Neurodegenerative Diseases
Source: Int J Mol Sci. 2026 Oct 29;27(21):9696. doi: 10.3390/ijms27219696 (PMC13763113; doi:10.3390/ijms27219696)
Supplement: Supplementary file 1 [file ijms-27-09696-s001.zip › Supplementary_Table_S1.pdf]

## Supplementary Table S1. PubMed search strategies and retrieval yields

Database: PubMed. Publication date range: 1 January 2015 to 6 September 2026. Language restriction: English. Deduplication procedure: Across the 13 PubMed searches, 3,083 records were retrieved. After removal of 622 duplicate records using PMIDs, 2,461 unique records remained. No article-type filter was applied at the database-search stage.

| Search category | Exact PubMed search string | Search field(s) | Date range | Filters | Records retrieved |
|---|---|---|---|---|---|
| Primary - Alzheimer's disease | ("Insulin-like Growth Factor-1"[All Fields] OR "IGF-1"[All Fields]) AND ("Alzheimer Disease"[MeSH Terms] OR "Alzheimer's disease"[All Fields]) AND english[lang] AND ("2015/01/01"[PDAT] : "2026/09/06"[PDAT]) | All Fields + MeSH Terms | 2015-01-01 to 2026-09-06 | English; no article-type filter | 304 |
| Primary - Parkinson's disease | ("Insulin-like Growth Factor-1"[All Fields] OR "IGF-1"[All Fields]) AND "Parkinson's disease"[All Fields] AND english[lang] AND ("2015/01/01"[PDAT] : "2026/09/06"[PDAT]) | All Fields | 2015-01-01 to 2026-09-06 | English; no article-type filter | 109 |
| Primary - Motor neuron disease / ALS | ("Insulin-like Growth Factor-1"[All Fields] OR "IGF-1"[All Fields] OR "IGF-I"[All Fields]) AND ("motor neuron disease"[All Fields] OR "amyotrophic lateral sclerosis"[All Fields]) AND english[lang] AND ("2015/01/01"[PDAT] : "2026/09/06"[PDAT]) | All Fields | 2015-01-01 to 2026-09-06 | English; no article-type filter | 50 |
| Primary - Huntington's disease | ("Insulin-like Growth Factor-1"[All Fields] OR "IGF-1"[All Fields] OR "IGF1"[All Fields]) AND ("Huntington Disease"[MeSH Terms] OR "Huntington's disease"[All Fields]) AND english[lang] AND ("2015/01/01"[PDAT] : "2026/09/06"[PDAT]) | All Fields + MeSH Terms | 2015-01-01 to 2026-09-06 | English; no article-type filter | 24 |
| Primary - Broader neurodegenerative disease | ("Insulin-like Growth Factor-1"[All Fields] OR "IGF-1"[All Fields] OR "IGF1"[All Fields]) AND ("neurodegenerative disease"[All Fields] OR "neurodegenerative diseases"[All Fields] OR "neurodegeneration"[All Fields]) AND english[lang] AND ("2015/01/01"[PDAT] : "2026/09/06"[PDAT]) | All Fields | 2015-01-01 to 2026-09-06 | English; no article-type filter | 410 |
| Supplementary - PI3K/Akt | ("Insulin-like Growth Factor-1"[All Fields] OR "IGF-1"[All Fields] OR "IGF1"[All Fields]) AND ("PI3K/Akt"[All Fields] OR "PI3K-Akt"[All Fields] OR "PI3K Akt"[All Fields]) AND english[lang] AND ("2015/01/01"[PDAT] : "2026/09/06"[PDAT]) | All Fields | 2015-01-01 to 2026-09-06 | English; no article-type filter | 1174 |
| Supplementary - Neuroinflammation | ("Insulin-like Growth Factor-1"[All Fields] OR "IGF-1"[All Fields] OR "IGF1"[All Fields]) AND "neuroinflammation"[All Fields] AND english[lang] AND ("2015/01/01"[PDAT] : "2026/09/06"[PDAT]) | All Fields | 2015-01-01 to 2026-09-06 | English; no article-type filter | 238 |
| Supplementary - Autophagy | ("Insulin-like Growth Factor-1"[All Fields] OR "IGF-1"[All Fields] OR "IGF1"[All Fields]) AND "autophagy"[All Fields] AND english[lang] AND ("2015/01/01"[PDAT] : "2026/09/06"[PDAT]) | All Fields | 2015-01-01 to 2026-09-06 | English; no article-type filter | 469 |
| Supplementary - Blood-brain barrier | ("Insulin-like Growth Factor-1"[All Fields] OR "IGF-1"[All Fields] OR "IGF1"[All Fields]) AND ("blood-brain barrier"[All Fields] OR "blood brain barrier"[All Fields]) AND english[lang] AND ("2015/01/01"[PDAT] : "2026/09/06"[PDAT]) | All Fields | 2015-01-01 to 2026-09-06 | English; no article-type filter | 103 |
| Supplementary - PTEN | ("Insulin-like Growth Factor-1"[All Fields] OR "IGF-1"[All Fields] OR "IGF1"[All Fields]) AND "PTEN"[All Fields] AND english[lang] AND ("2015/01/01"[PDAT] : "2026/09/06"[PDAT]) | All Fields | 2015-01-01 to 2026-09-06 | English; no article-type filter | 163 |
| Supplementary - SHIP2 | ("Insulin-like Growth Factor-1"[All Fields] OR "IGF-1"[All Fields] OR "IGF1"[All Fields]) AND ("SHIP2"[All Fields] OR "INPPL1"[All Fields]) AND english[lang] AND ("2015/01/01"[PDAT] : "2026/09/06"[PDAT]) | All Fields | 2015-01-01 to 2026-09-06 | English; no article-type filter | 2 |
| Supplementary - Intranasal delivery | ("Insulin-like Growth Factor-1"[All Fields] OR "IGF-1"[All Fields] OR "IGF1"[All Fields]) AND ("intranasal delivery"[All Fields] OR "intranasal administration"[All Fields]) AND english[lang] AND ("2015/01/01"[PDAT] : "2026/09/06"[PDAT]) | All Fields | 2015-01-01 to 2026-09-06 | English; no article-type filter | 28 |

| Search category | Exact PubMed search string | Search field(s) | Date range | Filters | Records retrieved |
|---|---|---|---|---|---|
| Supplementary - Nanocarrier | ("Insulin-like Growth Factor-1"[All Fields] OR "IGF-1"[All Fields] OR "IGF1"[All Fields]) AND ("nanocarrier"[All Fields] OR "nanocarriers"[All Fields]) AND english[lang] AND ("2015/01/01"[PDAT] : "2026/09/06"[PDAT]) | All Fields | 2015-01-01 to 2026-09-06 | English; no article-type filter | 9 |
