# Supplementary material for: Research Progress of Insulin-like Growth Factor-1 in Major Neurodegenerative Diseases
Source: Int J Mol Sci. 2026 Oct 29;27(21):9696. doi: 10.3390/ijms27219696 (PMC13763113; doi:10.3390/ijms27219696)
Supplement: Supplementary file 1 [file ijms-27-09696-s001.zip › Supplementary_Table_S2.pdf]

Supplementary Table S2. Reference-level list of literature cited in the narrative synthesis

Note: Reference numbering follows the revised manuscript. This table includes the 154 references cited in the revised manuscript; the 159 articles reported as retained after full-text screening in Section 7 constitute a separate screening dataset. Deduplication procedure: Across the 13 PubMed searches, 3,083 records were retrieved. After removal of 622 duplicate records using PMIDs, 2,461 unique records remained. References published before 2015 were retained for foundational context and are identified separately. The table is descriptive and does not imply that all cited articles were retrieved by a single PubMed query.

Ref.	First author (year)	Article title	Journal	Review section / topic	Inclusion basis	DOI
1	Teleanu (2022)	An Overview of Oxidative Stress, Neuroinflammation, and Neurodegenerative Diseases	Int J Mol Sci	General IGF-1/NDD background	2015–2026 literature included in narrative synthesis	10.3390/ijms23115938
2	Yang (2025)	Overcoming the Blood-Brain Barrier: Advanced Strategies in Targeted Drug Delivery for Neurodegenerative Diseases	Pharmaceutics	General IGF-1/NDD background	2015–2026 literature included in narrative synthesis	10.3390/pharmaceutics17081041
3	Cummings (2017)	Disease modification and Neuroprotection in neurodegenerative disorders	Transl Neurodegener	General IGF-1/NDD background	2015–2026 literature included in narrative synthesis	10.1186/s40035-017-0096-2
4	Nieto-Estévez (2016)	IGF-I: A Key Growth Factor that Regulates Neurogenesis and Synaptogenesis from Embryonic to Adult Stages of the Brain	Front Neurosci	General IGF-1/NDD background	2015–2026 literature included in narrative synthesis	10.3389/fnins.2016.00052
5	Aguiar-Oliveira (2026)	Growth Hormone and IGF-1 Actions in the Brain and Neuropsychiatric Diseases	Physiology (Bethesda)	General IGF-1/NDD background	2015–2026 literature included in narrative synthesis	10.1152/physiol.00009.2025
6	Ahmad (2020)	Implications of Insulin-Like Growth Factor-1 in Skeletal Muscle and Various Diseases	Cells	General IGF-1/NDD background	2015–2026 literature included in narrative synthesis	10.3390/cells9081773
7	Aguirre (2016)	Insulin-like growth factor-1 deficiency and metabolic syndrome	J Transl Med	General IGF-1/NDD background	2015–2026 literature included in narrative synthesis	10.1186/s12967-015-0762-z
8	Ji (2018)	IGF1 affects macrophage invasion and activation and TNF-α production in the sciatic nerves of female SOD1G93A mice	Neurosci Lett	General IGF-1/NDD background	2015–2026 literature included in narrative synthesis	10.1016/j.neulet.2017.12.062
9	Bailes (2021)	Insulin-Like Growth Factor-1 (IGF-1) and Its Monitoring in Medical Diagnostic and in Sports	Biomolecules	General IGF-1/NDD background	2015–2026 literature included in narrative synthesis	10.3390/biom11020217
10	Martín (2021)	IGF-1 and IGFBP-3 in Inflammatory Cachexia	Int J Mol Sci	General IGF-1/NDD background	2015–2026 literature included in narrative synthesis	10.3390/ijms22179469
11	Miao (2025)	Insulin-Like Growth Factor Signaling in Alzheimer's Disease: Pathophysiology and Therapeutic Strategies	Mol Neurobiol	General IGF-1/NDD background	2015–2026 literature included in narrative synthesis	10.1007/s12035-024-04457-1
12	Kaur (2025)	Uncovering the intricacies of IGF-1 in Alzheimer's disease: new insights from regulation to therapeutic targeting	Inflammopharmacology	General IGF-1/NDD background	2015–2026 literature included in narrative synthesis	10.1007/s10787-025-01641-0
13	Manni (2026)	Role of insulin-like growth factor-1/2 and related signaling in parkinson's disease	Metab Brain Dis	General IGF-1/NDD background	2015–2026 literature included in narrative synthesis	10.1007/s11011-026-01807-x
14	Cao (2023)	Circulating insulin-like growth factor-1 and brain health: Evidence from 369,711 participants in the UK Biobank	Alzheimers Res Ther	General IGF-1/NDD background	2015–2026 literature included in narrative synthesis	10.1186/s13195-023-01288-5
15	Hayes (2021)	Preclinical and clinical evidence of IGF-1 as a prognostic marker and acute intervention with ischemic stroke	J Cereb Blood Flow Metab	General IGF-1/NDD background	2015–2026 literature included in narrative synthesis	10.1177/0271678x211000894
16	Werner (2019)	Investigational IGF1R inhibitors in early stage clinical trials for cancer therapy	Expert Opin Investig Drugs	General IGF-1/NDD background	2015–2026 literature included in narrative synthesis	10.1080/13543784.2019.1694660
17	Arjunan (2023)	Identification of the molecular mechanism of insulin-like growth factor-1 (IGF-1): a promising therapeutic target for neurodegenerative diseases associated with metabolic syndrome	Cell Biosci	General IGF-1/NDD background	2015–2026 literature included in narrative synthesis	10.1186/s13578-023-00966-z
18	Ghazi Sherbaf (2018)	Serum Insulin-Like Growth Factor-1 in Parkinson's Disease; Study of Cerebrospinal Fluid Biomarkers and White Matter Microstructure	Front Endocrinol (Lausanne)	General IGF-1/NDD background	2015–2026 literature included in narrative synthesis	10.3389/fendo.2018.00608
19	Long (2019)	Alzheimer Disease: An Update on Pathobiology and Treatment Strategies	Cell	Cross-disease / growth-factor context	2015–2026 literature included in narrative synthesis	10.1016/j.cell.2019.09.001
20	Poewe (2017)	Parkinson disease	Nat Rev Dis Primers	Cross-disease / growth-factor context	2015–2026 literature included in narrative synthesis	10.1038/nrdp.2017.13

Ref.	First author (year)	Article title	Journal	Review section / topic	Inclusion basis	DOI
21	Kalia (2015)	Parkinson's disease	The Lancet	Cross-disease / growth-factor context	2015–2026 literature included in narrative synthesis	10.1016/S0140-6736(14)61393-3
22	Prasad (2019)	Molecular Mechanisms of TDP-43 Misfolding and Pathology in Amyotrophic Lateral Sclerosis	Front Mol Neurosci	Cross-disease / growth-factor context	2015–2026 literature included in narrative synthesis	10.3389/fnmol.2019.00025
23	Reiner (2018)	Disrupted striatal neuron inputs and outputs in Huntington's disease	CNS Neurosci Ther	Cross-disease / growth-factor context	2015–2026 literature included in narrative synthesis	10.1111/cns.12844
24	Wilson (2023)	Hallmarks of neurodegenerative diseases	Cell	Cross-disease / growth-factor context	2015–2026 literature included in narrative synthesis	10.1016/j.cell.2022.12.032
25	Bianchi (2017)	Neurotrophic and Neuroregenerative Effects of GH/IGF1	Int J Mol Sci	Cross-disease / growth-factor context	2015–2026 literature included in narrative synthesis	10.3390/ijms18112441
26	Wang (2024)	Alleviation of behavioral deficits, amyloid-β deposition, and mitochondrial structure damage associated with mitophagy upregulation in AD animal models via AAV9-IGF-1 treatment	Brain Res	Cross-disease / growth-factor context	2015–2026 literature included in narrative synthesis	10.1016/j.brainres.2023.148743
27	Castilla-Cortázar (2020)	Is insulin-like growth factor-1 involved in Parkinson's disease development?	J Transl Med	Cross-disease / growth-factor context	2015–2026 literature included in narrative synthesis	10.1186/s12967-020-02223-0
28	Pennuto (2020)	Insulin-like growth factor 1 signaling in motor neuron and polyglutamine diseases: From molecular pathogenesis to therapeutic perspectives	Front Neuroendocrinol	Cross-disease / growth-factor context	2015–2026 literature included in narrative synthesis	10.1016/j.yfrne.2020.100821
29	Naia (2016)	Insulin and IGF-1 regularize energy metabolites in neural cells expressing full-length mutant huntingtin	Neuropeptides	Cross-disease / growth-factor context	2015–2026 literature included in narrative synthesis	10.1016/j.npep.2016.01.009
30	Canu (2017)	The Intersection of NGF/TrkA Signaling and Amyloid Precursor Protein Processing in Alzheimer's Disease Neuropathology	Int J Mol Sci	Cross-disease / growth-factor context	2015–2026 literature included in narrative synthesis	10.3390/ijms18061319
31	Wang (2022)	BDNF signaling in context: From synaptic regulation to psychiatric disorders	Cell	Cross-disease / growth-factor context	2015–2026 literature included in narrative synthesis	10.1016/j.cell.2021.12.003
32	Gao (2022)	Brain-derived neurotrophic factor in Alzheimer's disease and its pharmaceutical potential	Transl Neurodegener	Cross-disease / growth-factor context	2015–2026 literature included in narrative synthesis	10.1186/s40035-022-00279-0
33	Geng (2023)	BDNF alleviates Parkinson's disease by promoting STAT3 phosphorylation and regulating neuronal autophagy	Cell Tissue Res	Cross-disease / growth-factor context	2015–2026 literature included in narrative synthesis	10.1007/s00441-023-03806-1
34	Scolz (2024)	Neuroprotection by ADAM10 inhibition requires TrkB signaling in the Huntington's disease hippocampus	Cell Mol Life Sci	Cross-disease / growth-factor context	2015–2026 literature included in narrative synthesis	10.1007/s00018-024-05382-1
35	Carrier-Ruiz (2025)	GDNF receptor GFRα1 is necessary for the maintenance of dopaminergic neurons in the adult substantia nigra	PLoS One	Cross-disease / growth-factor context	2015–2026 literature included in narrative synthesis	10.1371/journal.pone.0331369
36	Rhymes (2022)	Bimodal regulation of axonal transport by the GDNF-RET signalling axis in healthy and diseased motor neurons	Cell Death Dis	Cross-disease / growth-factor context	2015–2026 literature included in narrative synthesis	10.1038/s41419-022-05031-0
37	Khan (2025)	Molecular mechanisms of GDNF/GFRA1/RET and PI3K/AKT/ERK signaling interplay in neuroprotection: Therapeutic strategies for treating neurological disorders	Neuropeptides	Cross-disease / growth-factor context	2015–2026 literature included in narrative synthesis	10.1016/j.npep.2025.102516
38	Liang (2026)	Electroacupuncture Improves the Learning and Memory by Modulating Hippocampal Glucose Metabolism through IGF1/IGF1R Signaling in Alzheimer's Disease	Adv Sci (Weinh)	Cross-disease / growth-factor context	2015–2026 literature included in narrative synthesis	10.1002/advs.202514241
39	Huang (2024)	Targeting the RUNX3-miR-186-3p-DAT-IGF1R axis as a therapeutic strategy in a Parkinson's disease model	J Transl Med	Cross-disease / growth-factor context	2015–2026 literature included in narrative synthesis	10.1186/s12967-024-05535-7
40	Fellows (2020)	IGF1R regulates retrograde axonal transport of signalling endosomes in motor neurons	EMBO Rep	Cross-disease / growth-factor context	2015–2026 literature included in narrative synthesis	10.15252/embr.201949129
41	Skotte (2020)	Compromised IGF signaling causes caspase-6 activation in Huntington disease	Exp Neurol	Cross-disease / growth-factor context	2015–2026 literature included in narrative synthesis	10.1016/j.expneurol.2020.113396
42	García-Huerta (2020)	Insulin-like growth factor 2 (IGF2) protects against Huntington's disease through the extracellular disposal of protein aggregates	Acta Neuropathol	Cross-disease / growth-factor context	2015–2026 literature included in narrative synthesis	10.1007/s00401-020-02183-1

Ref.	First author (year)	Article title	Journal	Review section / topic	Inclusion basis	DOI
43	Emery (2026)	New perspectives on VEGF signalling in Alzheimer's disease	Brain Pathol	Cross-disease / growth-factor context	2015–2026 literature included in narrative synthesis	10.1111/bpa.70100
44	Silva-Hucha (2024)	VEGF expression disparities in brainstem motor neurons of the SOD1(G93A) ALS model: Correlations with neuronal vulnerability	Neurotherapeutics	Cross-disease / growth-factor context	2015–2026 literature included in narrative synthesis	10.1016/j.neurot.2024.e00340
45	Zimbone (2025)	VEGF and the VEGF(73-101) Fragment Prevent MPP(+) Induced Mitochondrial Dysfunction in a Cell Model of Parkinson's Disease	Mol Neurobiol	Cross-disease / growth-factor context	2015–2026 literature included in narrative synthesis	10.1007/s12035-025-05213-9
46	Wang (2026)	FGF-FGFR Signaling in Parkinson's Disease: Mechanistic Links to Ferroptosis and Neuroprotection	Brain Sci	Cross-disease / growth-factor context	2015–2026 literature included in narrative synthesis	10.3390/brainsci16020151
47	Chen (2026)	FGF22/FGFR2/YAP modulates ferroptosis to suppress neurodegeneration and cognitive impairment in Alzheimer's disease	Exp Neurol	Cross-disease / growth-factor context	2015–2026 literature included in narrative synthesis	10.1016/j.expneurol.2025.115630
48	Pasquin (2015)	Ciliary neurotrophic factor (CNTF): New facets of an old molecule for treating neurodegenerative and metabolic syndrome pathologies	Cytokine Growth Factor Rev	Cross-disease / growth-factor context	2015–2026 literature included in narrative synthesis	10.1016/j.cytogfr.2015.07.007
49	You (2026)	Involvement of 75NTR extracellular domain in rotenone-induced Parkinson's disease cell models	Neuroreport	Cross-disease / growth-factor context	2015–2026 literature included in narrative synthesis	10.1097/wnr.0000000000002258
50	Ali (2024)	The Molecular Pathway of p75 Neurotrophin Receptor (p75NTR) in Parkinson's Disease: The Way of New Inroads	Mol Neurobiol	Cross-disease / growth-factor context	2015–2026 literature included in narrative synthesis	10.1007/s12035-023-03727-8
51	Huang (2026)	MerTK-triggered TGFβ1 autocrine signal regulates microglial response to neurodegeneration	Nat Commun	Cross-disease / growth-factor context	2015–2026 literature included in narrative synthesis	10.1038/s41467-026-69189-3
52	Karampetsou (2022)	The promise of the TGF-β superfamily as a therapeutic target for Parkinson's disease	Neurobiol Dis	Cross-disease / growth-factor context	2015–2026 literature included in narrative synthesis	10.1016/j.nbd.2022.105805
53	Lee (2024)	Dual effects of TGF-β inhibitor in ALS - inhibit contracture and neurodegeneration	J Neurochem	Cross-disease / growth-factor context	2015–2026 literature included in narrative synthesis	10.1111/jnc.16102
54	Yu (2026)	A new approach for treating AD: Guifu Dihuang Pills improves brain insulin resistance by promoting NrCAM to activate the EGFR/PI3K/Akt signaling pathway	J Ethnopharmacol	Cross-disease / growth-factor context	2015–2026 literature included in narrative synthesis	10.1016/j.jep.2025.120642
55	Iwakura (2005)	Influences of dopaminergic lesion on epidermal growth factor-ErbB signals in Parkinson's disease and its model: neurotrophic implication in nigrostriatal neurons	J Neurochem	Cross-disease / growth-factor context	Pre-2015 foundational reference	10.1111/j.1471-4159.2005.03073.x
56	Cunnane (2020)	Brain energy rescue: an emerging therapeutic concept for neurodegenerative disorders of ageing	Nat Rev Drug Discov	Alzheimer's disease (Section 3)	2015–2026 literature included in narrative synthesis	10.1038/s41573-020-0072-x
57	Scheltens (2021)	Alzheimer's disease	Lancet	Alzheimer's disease (Section 3)	2015–2026 literature included in narrative synthesis	10.1016/s0140-6736(20)32205-4
58	de la Monte (2017)	Insulin Resistance and Neurodegeneration: Progress Towards the Development of New Therapeutics for Alzheimer's Disease	Drugs	Alzheimer's disease (Section 3)	2015–2026 literature included in narrative synthesis	10.1007/s40265-016-0674-0
59	Rudman (2026)	Recent advances in Alzheimer's disease: From molecular mechanisms to therapeutic strategies	Cell	Alzheimer's disease (Section 3)	2015–2026 literature included in narrative synthesis	10.1016/j.cell.2026.06.006
60	Ivanova (2025)	Alzheimer's Disease: From Molecular Mechanisms to Promising Therapeutic Strategies	Int J Mol Sci	Alzheimer's disease (Section 3)	2015–2026 literature included in narrative synthesis	10.3390/ijms26199444
61	Singh (2025)	Amyloid-β disrupts APP-regulated protein aggregation and dissociation from recycling endosomal membranes	Embo j	Alzheimer's disease (Section 3)	2015–2026 literature included in narrative synthesis	10.1038/s44318-025-00497-y
62	Ruan (2021)	Alzheimer's disease brain-derived extracellular vesicles spread tau pathology in interneurons	Brain	Alzheimer's disease (Section 3)	2015–2026 literature included in narrative synthesis	10.1093/brain/awaa376
63	Pîrşcoveanu (2017)	Tau protein in neurodegenerative diseases - a review	Rom J Morphol Embryol	Alzheimer's disease (Section 3)	2015–2026 literature included in narrative synthesis	
64	Pedrolli (2026)	Growth hormone-releasing hormone attenuates amyloid deposition and neuroinflammation in Alzheimer's disease models	Cell Death Dis	Alzheimer's disease (Section 3)	2015–2026 literature included in narrative synthesis	10.1038/s41419-026-08699-w

Ref.	First author (year)	Article title	Journal	Review section / topic	Inclusion basis	DOI
65	Nam (2025)	Dual modulation of amyloid beta and tau aggregation and dissociation in Alzheimer's disease: a comprehensive review of the characteristics and therapeutic strategies	Transl Neurodegener	Alzheimer's disease (Section 3)	2015–2026 literature included in narrative synthesis	10.1186/s40035-025-00479-4
66	Ng (2024)	Tau depletion in human neurons mitigates Aβ-driven toxicity	Mol Psychiatry	Alzheimer's disease (Section 3)	2015–2026 literature included in narrative synthesis	10.1038/s41380-024-02463-2
67	Kellar (2020)	Brain insulin resistance in Alzheimer's disease and related disorders: mechanisms and therapeutic approaches	Lancet Neurol	Alzheimer's disease (Section 3)	2015–2026 literature included in narrative synthesis	10.1016/s1474-4422(20)30231-3
68	Blázquez (2022)	Significance of Brain Glucose Hypometabolism, Altered Insulin Signal Transduction, and Insulin Resistance in Several Neurological Diseases	Front Endocrinol (Lausanne)	Alzheimer's disease (Section 3)	2015–2026 literature included in narrative synthesis	10.3389/fendo.2022.873301
69	Chapple (2025)	Type 3 Diabetes: Linking Insulin Resistance to Cognitive Decline	Diseases	Alzheimer's disease (Section 3)	2015–2026 literature included in narrative synthesis	10.3390/diseases13110359
70	Selles (2020)	Adenovirus-Mediated Transduction of Insulin-Like Growth Factor 1 Protects Hippocampal Neurons from the Toxicity of Aβ Oligomers and Prevents Memory Loss in an Alzheimer Mouse Model	Mol Neurobiol	Alzheimer's disease (Section 3)	2015–2026 literature included in narrative synthesis	10.1007/s12035-019-01827-y
71	Zhang (2013)	Alternations of central insulin-like growth factor-1 sensitivity in APP/PS1 transgenic mice and neuronal models	J Neurosci Res	Alzheimer's disease (Section 3)	Pre-2015 foundational reference	10.1002/jnr.23201
72	Wen (2023)	Regulation of Phosphoinositide Signaling by Scaffolds at Cytoplasmic Membranes	Biomolecules	Alzheimer's disease (Section 3)	2015–2026 literature included in narrative synthesis	10.3390/biom13091297
73	Desale (2021)	Phosphoinositides signaling modulates microglial actin remodeling and phagocytosis in Alzheimer's disease	Cell Commun Signal	Alzheimer's disease (Section 3)	2015–2026 literature included in narrative synthesis	10.1186/s12964-021-00715-0
74	Chu (2021)	Dysregulated phosphoinositide 3-kinase signaling in microglia: shaping chronic neuroinflammation	J Neuroinflammation	Alzheimer's disease (Section 3)	2015–2026 literature included in narrative synthesis	10.1186/s12974-021-02325-6
75	Kalous (2023)	A Role of PI3K/Akt Signaling in Oocyte Maturation and Early Embryo Development	Cells	Alzheimer's disease (Section 3)	2015–2026 literature included in narrative synthesis	10.3390/cells12141830
76	Wijayaratna (2023)	The spatial distribution of GPCR and Gβγ activity across a cell dictates PIP3 dynamics	Sci Rep	Alzheimer's disease (Section 3)	2015–2026 literature included in narrative synthesis	10.1038/s41598-023-29639-0
77	John (2023)	The Role of C2 Domains in Two Different Phosphatases: PTEN and SHIP2	Membranes (Basel)	Alzheimer's disease (Section 3)	2015–2026 literature included in narrative synthesis	10.3390/membranes13040408
78	Dempsey (2021)	The structural basis of PTEN regulation by multi-site phosphorylation	Nat Struct Mol Biol	Alzheimer's disease (Section 3)	2015–2026 literature included in narrative synthesis	10.1038/s41594-021-00668-5
79	Waddell (2023)	Mechanisms controlling membrane recruitment and activation of the autoinhibited SHIP1 inositol 5-phosphatase	J Biol Chem	Alzheimer's disease (Section 3)	2015–2026 literature included in narrative synthesis	10.1016/j.jbc.2023.105022
80	Cabral-Dias (2023)	Control of phosphatidylinositol-3-kinase signaling by nanoscale membrane compartmentalization	Bioessays	Alzheimer's disease (Section 3)	2015–2026 literature included in narrative synthesis	10.1002/bies.202200196
81	Liu (2022)	PTEN Dual Lipid- and Protein-Phosphatase Function in Tumor Progression	Cancers (Basel)	Alzheimer's disease (Section 3)	2015–2026 literature included in narrative synthesis	10.3390/cancers14153666
82	Michael (2021)	The 5-Phosphatase SHIP2 Promotes Neutrophil Chemotaxis and Recruitment	Front Immunol	Alzheimer's disease (Section 3)	2015–2026 literature included in narrative synthesis	10.3389/fimmu.2021.671756
83	Olufunmilayo (2023)	INPP5D/SHIP1: Expression, Regulation and Roles in Alzheimer's Disease Pathophysiology	Genes (Basel)	Alzheimer's disease (Section 3)	2015–2026 literature included in narrative synthesis	10.3390/genes14101845
84	Rangan (2022)	Fasting-mimicking diet cycles reduce neuroinflammation to attenuate cognitive decline in Alzheimer's models	Cell Rep	Alzheimer's disease (Section 3)	2015–2026 literature included in narrative synthesis	10.1016/j.celrep.2022.111417
85	Costantini (2005)	A TrkA-to-p75NTR molecular switch activates amyloid beta-peptide generation during aging	Biochem J	Alzheimer's disease (Section 3)	Pre-2015 foundational reference	10.1042/bj20050700
86	Costantini (2006)	An aging pathway controls the TrkA to p75NTR receptor switch and amyloid beta-peptide generation	Embo j	Alzheimer's disease (Section 3)	Pre-2015 foundational reference	10.1038/sj.emboj.7601062
87	Bruno (2023)	The Nerve Growth Factor Receptor (NGFR/p75(NTR)): A Major Player in Alzheimer's Disease	Int J Mol Sci	Alzheimer's disease (Section 3)	2015–2026 literature included in narrative synthesis	10.3390/ijms24043200

Ref.	First author (year)	Article title	Journal	Review section / topic	Inclusion basis	DOI
88	Kropf (2023)	Age-induced nitrative stress decreases retrograde transport of proNGF via TrkA and increases proNGF retrograde transport and neurodegeneration via p75(NTR)	Front Mol Neurosci	Alzheimer's disease (Section 3)	2015–2026 literature included in narrative synthesis	10.3389/fnmol.2023.1241420
89	Puglielli (2008)	Aging of the brain, neurotrophin signaling, and Alzheimer's disease: is IGF1-R the common culprit?	Neurobiol Aging	Alzheimer's disease (Section 3)	Pre-2015 foundational reference	10.1016/j.neurobiolaging.2007.01.010
90	Jiao (2022)	Amyloidogenesis and Neurotrophic Dysfunction in Alzheimer's Disease: Do They have a Common Regulating Pathway?	Cells	Alzheimer's disease (Section 3)	2015–2026 literature included in narrative synthesis	10.3390/cells11203201
91	Fronza (2023)	The neurobiology and therapeutic potential of multi-targeting β-secretase, glycogen synthase kinase 3β and acetylcholinesterase in Alzheimer's disease	Ageing Res Rev	Alzheimer's disease (Section 3)	2015–2026 literature included in narrative synthesis	10.1016/j.arr.2023.102033
92	Li (2009)	Egr-1 and Hipk2 are required for the TrkA to p75(NTR) switch that occurs downstream of IGF1-R	Neurobiol Aging	Alzheimer's disease (Section 3)	Pre-2015 foundational reference	10.1016/j.neurobiolaging.2008.02.015
93	Kumar (2022)	Implications of Phosphoinositide 3-Kinase-Akt (PI3K-Akt) Pathway in the Pathogenesis of Alzheimer's Disease	Mol Neurobiol	Alzheimer's disease (Section 3)	2015–2026 literature included in narrative synthesis	10.1007/s12035-021-02611-7
94	Sanjai (2026)	Molecular neurobiological insights on integrative role of Trk neurotrophic receptors and their small-molecule agonists in neurodegenerative conditions	Exp Brain Res	Alzheimer's disease (Section 3)	2015–2026 literature included in narrative synthesis	10.1007/s00221-026-07327-5
95	Gabbouj (2019)	Altered Insulin Signaling in Alzheimer's Disease Brain - Special Emphasis on PI3K-Akt Pathway	Front Neurosci	Alzheimer's disease (Section 3)	2015–2026 literature included in narrative synthesis	10.3389/fnins.2019.00629
96	Zhang (2024)	Recent advances in Alzheimer's disease: Mechanisms, clinical trials and new drug development strategies	Signal Transduct Target Ther	Alzheimer's disease (Section 3)	2015–2026 literature included in narrative synthesis	10.1038/s41392-024-01911-3
97	Ito (2012)	Involvement of insulin-like growth factor 1 receptor signaling in the amyloid-β peptide oligomers-induced p75 neurotrophin receptor protein expression in mouse hippocampus	J Alzheimers Dis	Alzheimer's disease (Section 3)	Pre-2015 foundational reference	10.3233/jad-2012-120046
98	Narayan (2025)	Reduced IGF-1 signaling fails to limit Alzheimer's disease progression in a novel rat model of IGF-1R haploinsufficiency	Sci Rep	Alzheimer's disease (Section 3)	2015–2026 literature included in narrative synthesis	10.1038/s41598-025-31601-1
99	Lauretti (2020)	Glycogen synthase kinase-3 signaling in Alzheimer's disease	Biochim Biophys Acta Mol Cell Res	Alzheimer's disease (Section 3)	2015–2026 literature included in narrative synthesis	10.1016/j.bbamcr.2020.118664
100	Sayas (2021)	GSK-3 and Tau: A Key Duet in Alzheimer's Disease	Cells	Alzheimer's disease (Section 3)	2015–2026 literature included in narrative synthesis	10.3390/cells10040721
101	Liu (2026)	Three-dimensional interactive network: Mitochondrial-metabolic-calcium homeostasis driving Alzheimer's disease	Genes Dis	Alzheimer's disease (Section 3)	2015–2026 literature included in narrative synthesis	10.1016/j.gendis.2025.101846
102	Ando (2021)	Dysregulation of Phosphoinositide 5-Phosphatases and Phosphoinositides in Alzheimer's Disease	Front Neurosci	Alzheimer's disease (Section 3)	2015–2026 literature included in narrative synthesis	10.3389/fnins.2021.614855
103	Pedicone (2020)	Pan-SHIP1/2 inhibitors promote microglia effector functions essential for CNS homeostasis	J Cell Sci	Alzheimer's disease (Section 3)	2015–2026 literature included in narrative synthesis	10.1242/jcs.238030
104	Feraco (2021)	Imaging of Substantia Nigra in Parkinson's Disease: A Narrative Review	Brain Sci	Parkinson's disease (Section 4)	2015–2026 literature included in narrative synthesis	10.3390/brainsci11060769
105	Hall (2014)	Hippocampal Lewy pathology and cholinergic dysfunction are associated with dementia in Parkinson's disease	Brain	Parkinson's disease (Section 4)	Pre-2015 foundational reference	10.1093/brain/awu193
106	Lin (2023)	Nigral Pathology Contributes to Microstructural Integrity of Striatal and Frontal Tracts in Parkinson's Disease	Mov Disord	Parkinson's disease (Section 4)	2015–2026 literature included in narrative synthesis	10.1002/mds.29510
107	Hayes (2019)	Parkinson's Disease and Parkinsonism	Am J Med	Parkinson's disease (Section 4)	2015–2026 literature included in narrative synthesis	10.1016/j.amjmed.2019.03.001
108	Sivaranjini (2021)	Morphological analysis of subcortical structures for assessment of cognitive dysfunction in Parkinson's disease using multi-atlas based segmentation	Cogn Neurodyn	Parkinson's disease (Section 4)	2015–2026 literature included in narrative synthesis	10.1007/s11571-021-09671-4
109	Pellecchia (2014)	Insulin-like growth factor-1 predicts cognitive functions at 2-year follow-up in early, drug-naïve Parkinson's disease	Eur J Neurol	Parkinson's disease (Section 4)	Pre-2015 foundational reference	10.1111/ene.12137

Ref.	First author (year)	Article title	Journal	Review section / topic	Inclusion basis	DOI
110	Saredakis (2019)	Conversion to MCI and dementia in Parkinson's disease: a systematic review and meta-analysis	Parkinsonism Relat Disord	Parkinson's disease (Section 4)	2015–2026 literature included in narrative synthesis	10.1016/j.parkreldis.2019.04.020
111	Rački (2022)	Cognitive Impact of Deep Brain Stimulation in Parkinson's Disease Patients: A Systematic Review	Front Hum Neurosci	Parkinson's disease (Section 4)	2015–2026 literature included in narrative synthesis	10.3389/fnhum.2022.867055
112	Zhu (2022)	Cortical atrophy is associated with cognitive impairment in Parkinson's disease: a combined analysis of cortical thickness and functional connectivity	Brain Imaging Behav	Parkinson's disease (Section 4)	2015–2026 literature included in narrative synthesis	10.1007/s11682-022-00714-w
113	Jellinger (2022)	Morphological differences between dementia with Lewy bodies and Parkinson's disease-dementia	Neuropathol Appl Neurobiol	Parkinson's disease (Section 4)	2015–2026 literature included in narrative synthesis	10.1111/nan.12708
114	He (2018)	Recent Advances in Biomarkers for Parkinson's Disease	Front Aging Neurosci	Parkinson's disease (Section 4)	2015–2026 literature included in narrative synthesis	10.3389/fnagi.2018.00305
115	Shi (2023)	Correlation between serum IGF-1 and EGF levels and neuropsychiatric and cognitive in Parkinson's disease patients	Neurol Sci	Parkinson's disease (Section 4)	2015–2026 literature included in narrative synthesis	10.1007/s10072-022-06490-1
116	Cilento (2019)	Mass spectrometry: A platform for biomarker discovery and validation for Alzheimer's and Parkinson's diseases	J Neurochem	Parkinson's disease (Section 4)	2015–2026 literature included in narrative synthesis	10.1111/jnc.14635
117	Kim (2018)	IGF-1 protects SH-SY5Y cells against MPP(+)-induced apoptosis via PI3K/PDK-1/Akt pathway	Endocr Connect	Parkinson's disease (Section 4)	2015–2026 literature included in narrative synthesis	10.1530/ec-17-0350
118	Wang (2020)	IGF-1 inhibits MPTP/MPP(+)-induced autophagy on dopaminergic neurons through the IGF-1R/PI3K-Akt-mTOR pathway and GPER	Am J Physiol Endocrinol Metab	Parkinson's disease (Section 4)	2015–2026 literature included in narrative synthesis	10.1152/ajpendo.00071.2020
119	Jęśko (2020)	Dysfunctional proteins in neuropsychiatric disorders: From neurodegeneration to autism spectrum disorders	Neurochem Int	Parkinson's disease (Section 4)	2015–2026 literature included in narrative synthesis	10.1016/j.neuint.2020.104853
120	Vidović (2022)	Alpha-Synuclein Aggregation Pathway in Parkinson's Disease: Current Status and Novel Therapeutic Approaches	Cells	Parkinson's disease (Section 4)	2015–2026 literature included in narrative synthesis	10.3390/cells11111732
121	Arawaka (2017)	Mechanisms underlying extensive Ser129-phosphorylation in α-synuclein aggregates	Acta Neuropathol Commun	Parkinson's disease (Section 4)	2015–2026 literature included in narrative synthesis	10.1186/s40478-017-0452-6
122	Khan (2023)	Enhanced mTORC1 signaling and protein synthesis in pathologic α-synuclein cellular and animal models of Parkinson's disease	Sci Transl Med	Parkinson's disease (Section 4)	2015–2026 literature included in narrative synthesis	10.1126/scitranslmed.add0499
123	Kang (2017)	TrkB neurotrophic activities are blocked by α-synuclein, triggering dopaminergic cell death in Parkinson's disease	Proc Natl Acad Sci U S A	Parkinson's disease (Section 4)	2015–2026 literature included in narrative synthesis	10.1073/pnas.1713969114
124	Gao (2015)	Alpha-synuclein overexpression negatively regulates insulin receptor substrate 1 by activating mTORC1/S6K1 signaling	Int J Biochem Cell Biol	Parkinson's disease (Section 4)	2015–2026 literature included in narrative synthesis	10.1016/j.biocel.2015.03.006
125	Yang (2018)	The Role of Insulin/IGF-1/PI3K/Akt/GSK3β Signaling in Parkinson's Disease Dementia	Front Neurosci	Parkinson's disease (Section 4)	2015–2026 literature included in narrative synthesis	10.3389/fnins.2018.00073
126	El Hajjar (2024)	Regulation of Glycogen Synthase Kinase-3β by Phosphorylation and O-β-Linked N-Acetylglucosaminylation: Implications on Tau Protein Phosphorylation	Biochemistry	Parkinson's disease (Section 4)	2015–2026 literature included in narrative synthesis	10.1021/acs.biochem.4c00095
127	Yu (2023)	The role of glycogen synthase kinase 3 beta in neurodegenerative diseases	Front Mol Neurosci	Parkinson's disease (Section 4)	2015–2026 literature included in narrative synthesis	10.3389/fnmol.2023.1209703
128	Credle (2015)	GSK-3β dysregulation contributes to parkinson's-like pathophysiology with associated region-specific phosphorylation and accumulation of tau and α-synuclein	Cell Death Differ	Parkinson's disease (Section 4)	2015–2026 literature included in narrative synthesis	10.1038/cdd.2014.179
129	Karampetsou (2017)	Phosphorylated exogenous alpha-synuclein fibrils exacerbate pathology and induce neuronal dysfunction in mice	Sci Rep	Parkinson's disease (Section 4)	2015–2026 literature included in narrative synthesis	10.1038/s41598-017-15813-8
130	Ayadi (2016)	IGF-1 protects dopamine neurons against oxidative stress: association with changes in phosphokinases	Exp Brain Res	Parkinson's disease (Section 4)	2015–2026 literature included in narrative synthesis	10.1007/s00221-016-4572-1
131	Kiriyama (2015)	The Function of Autophagy in Neurodegenerative Diseases	Int J Mol Sci	Parkinson's disease (Section 4)	2015–2026 literature included in narrative synthesis	10.3390/ijms161125990

Ref.	First author (year)	Article title	Journal	Review section / topic	Inclusion basis	DOI
132	Alers (2012)	Role of AMPK-mTOR-Ulk1/2 in the regulation of autophagy: cross talk, shortcuts, and feedbacks	Mol Cell Biol	Parkinson's disease (Section 4)	Pre-2015 foundational reference	10.1128/mcb.06159-11
133	O'Brien (2024)	New developments in the diagnosis and management of motor neuron disease	Br Med Bull	MND/ALS (Section 5)	2015–2026 literature included in narrative synthesis	10.1093/bmb/ldae010
134	Wen (2026)	Latest progress and challenges in drug development for degenerative motor neuron diseases	Neural Regen Res	MND/ALS (Section 5)	2015–2026 literature included in narrative synthesis	10.4103/nrr.Nrr-d-24-01266
135	The (2024)	Motor neuron disease: improving quality of life for patients	Lancet	MND/ALS (Section 5)	2015–2026 literature included in narrative synthesis	10.1016/s0140-6736(24)00972-3
136	Brennan (2022)	Motor neuron disease: The last 12 months	Aust J Gen Pract	MND/ALS (Section 5)	2015–2026 literature included in narrative synthesis	10.31128/ajgp-07-21-6097
137	Nagel (2020)	Association of Insulin-like Growth Factor 1 Concentrations with Risk for and Prognosis of Amyotrophic Lateral Sclerosis - Results from the ALS Registry Swabia	Sci Rep	MND/ALS (Section 5)	2015–2026 literature included in narrative synthesis	10.1038/s41598-020-57744-x
138	De Leon-Oliva (2023)	AIF1: Function and Connection with Inflammatory Diseases	Biology (Basel)	MND/ALS (Section 5)	2015–2026 literature included in narrative synthesis	10.3390/biology12050694
139	Lituma (2021)	Altered synaptic connectivity and brain function in mice lacking microglial adapter protein Iba1	Proc Natl Acad Sci U S A	MND/ALS (Section 5)	2015–2026 literature included in narrative synthesis	10.1073/pnas.2115539118
140	Yoshida (2020)	Mechanisms of IGF-1-Mediated Regulation of Skeletal Muscle Hypertrophy and Atrophy	Cells	MND/ALS (Section 5)	2015–2026 literature included in narrative synthesis	10.3390/cells9091970
141	Bonifacio (2017)	IGF-1 prevents simvastatin-induced myotoxicity in C2C12 myotubes	Arch Toxicol	MND/ALS (Section 5)	2015–2026 literature included in narrative synthesis	10.1007/s00204-016-1871-z
142	Adachi (2019)	Insulin-Like Growth Factor-I Protects Against the Detrimental Effects of Advanced Glycation End Products and High Glucose in Myoblastic C2C12 Cells	Calcif Tissue Int	MND/ALS (Section 5)	2015–2026 literature included in narrative synthesis	10.1007/s00223-019-00537-w
143	Dobrowolny (2005)	Muscle expression of a local Igf-1 isoform protects motor neurons in an ALS mouse model	J Cell Biol	MND/ALS (Section 5)	Pre-2015 foundational reference	10.1083/jcb.200407021
144	Troncoso-Escudero (2020)	On the Right Track to Treat Movement Disorders: Promising Therapeutic Approaches for Parkinson's and Huntington's Disease	Front Aging Neurosci	Huntington's disease (Section 6)	2015–2026 literature included in narrative synthesis	10.3389/fnagi.2020.571185
145	Stoker (2022)	Huntington's disease: diagnosis and management	Pract Neurol	Huntington's disease (Section 6)	2015–2026 literature included in narrative synthesis	10.1136/practneurol-2021-003074
146	Humbert (2002)	The IGF-1/Akt pathway is neuroprotective in Huntington's disease and involves Huntingtin phosphorylation by Akt	Dev Cell	Huntington's disease (Section 6)	Pre-2015 foundational reference	10.1016/s1534-5807(02)00188-0
147	Escartin (2004)	Insulin growth factor-1 protects against excitotoxicity in the rat striatum	Neuroreport	Huntington's disease (Section 6)	Pre-2015 foundational reference	10.1097/00001756-200410050-00022
148	Lopes (2014)	IGF-1 intranasal administration rescues Huntington's disease phenotypes in YAC128 mice	Mol Neurobiol	Huntington's disease (Section 6)	Pre-2015 foundational reference	10.1007/s12035-013-8585-5
149	Escartin (2007)	IGF-1 exacerbates the neurotoxicity of the mitochondrial inhibitor 3NP in rats	Neurosci Lett	Huntington's disease (Section 6)	Pre-2015 foundational reference	10.1016/j.neulet.2007.08.031
150	Salem (2016)	Insulin-Like Growth Factor-1 but Not Insulin Predicts Cognitive Decline in Huntington's Disease	PLoS One	Huntington's disease (Section 6)	2015–2026 literature included in narrative synthesis	10.1371/journal.pone.0162890
151	Kratter (2016)	Serine 421 regulates mutant huntingtin toxicity and clearance in mice	J Clin Invest	Huntington's disease (Section 6)	2015–2026 literature included in narrative synthesis	10.1172/jci80339
152	Xu (2020)	pS421 huntingtin modulates mitochondrial phenotypes and confers neuroprotection in an HD hiPSC model	Cell Death Dis	Huntington's disease (Section 6)	2015–2026 literature included in narrative synthesis	10.1038/s41419-020-02983-z
153	Bryan (2020)	Manganese Acts upon Insulin/IGF Receptors to Phosphorylate AKT and Increase Glucose Uptake in Huntington's Disease Cells	Mol Neurobiol	Huntington's disease (Section 6)	2015–2026 literature included in narrative synthesis	10.1007/s12035-019-01824-1
154	Tung (2025)	Insulin-like growth factor 2 reduces Huntington's disease aggregates via AKT and NF-κB signaling in huntington's disease	Cell Biosci	Huntington's disease (Section 6)	2015–2026 literature included in narrative synthesis	10.1186/s13578-025-01452-4
